# Correction: children in the household and risk of severe COVID-19 during the first three waves of the pandemic: a prospective registry-based cohort study of 1.5 million swedish men

**DOI:** 10.1136/bmjopen-2022-063640corr1

**Published:** 2025-11-12

 af Geijerstam A, Mehlig K, Hunsberger M, *et al*. Children in the household and risk of severe COVID-19 during the first three waves of the pandemic: a prospective registry-based cohort study of 1.5 million Swedish men. *BMJ Open* 2022;12:e063640. doi:10.1136/bmjopen-2022-063640

This article was previously published with an error.

An error in figure 3 has been identified, where the estimates were misplaced. The figure has been corrected in the paper and is provided here for reference. Also, the online supplemental file has been updated. These corrections do not affect the overall results or conclusions.

**Figure 3:**
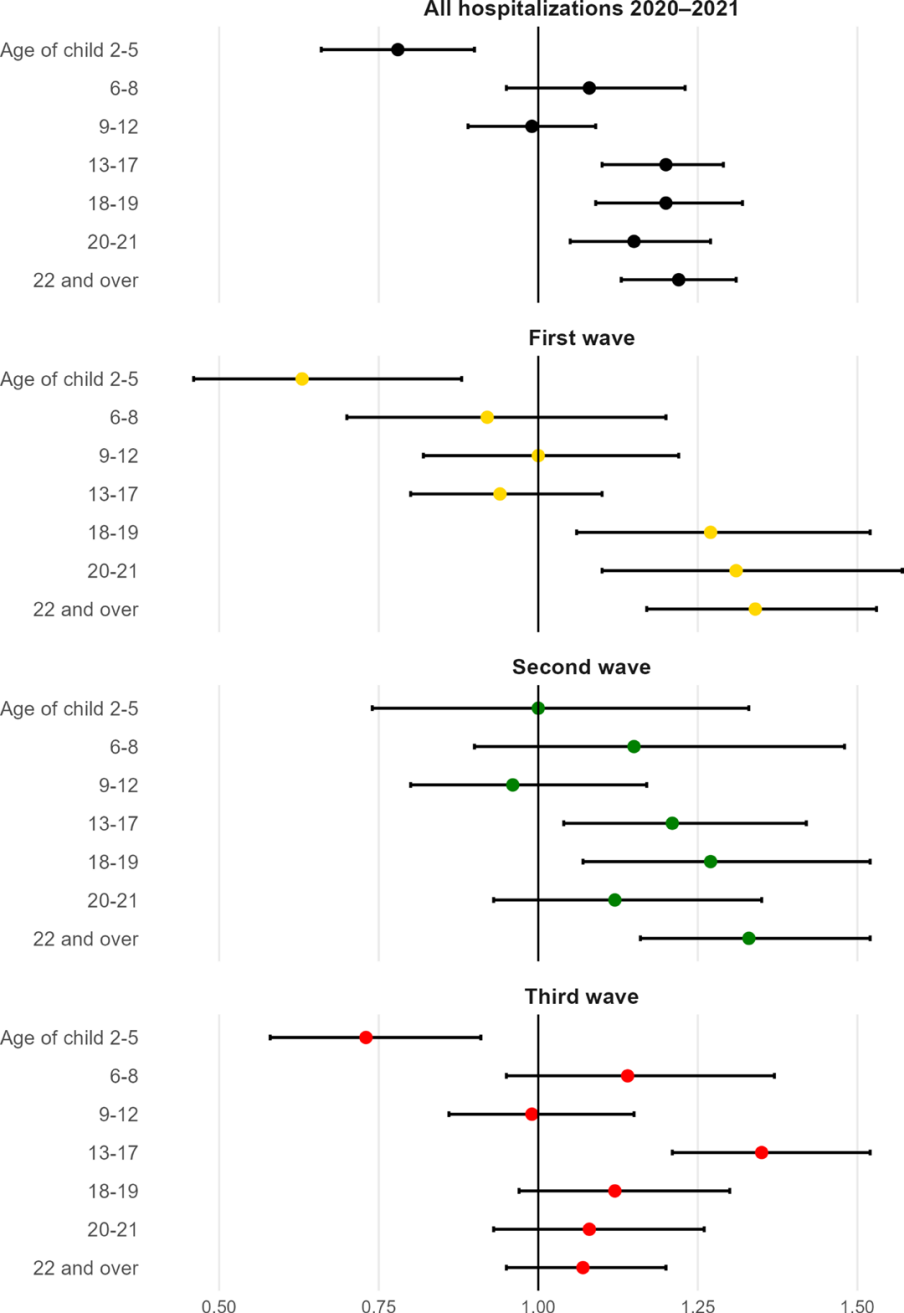
Associations between children in the household and hospitalisation due to COVID-19 (n=1 557 061). ORs with 95% CI. Model controlled for children in other age groups, age, baseline BMI, CRF, height, chronic morbidity, parental education, income, profession and place of residence in 2018. BMI, body mass index; CRF, cardiorespiratory fitness.

## Supplementary material

10.1136/bmjopen-2022-063640corr1 online supplemental file 1

