# Supplementary material for: Children in the household and risk of severe COVID-19 during the first three waves of the pandemic: a prospective registry-based cohort study of 1.5 million Swedish men
Source: BMJ Open. 2022 Aug 11;12(8):e063640. doi: 10.1136/bmjopen-2022-063640 (PMC9378946; doi:10.1136/bmjopen-2022-063640)
Supplement: online supplemental file 1 [file bmjopen-12-8-s001.pdf]

Table S1. Included main diagnoses divided by categories when COVID-19 is secondary diagnosis.

Included categories	ICD-codes
**COVID-related symptoms**	
Cough	R05
Abnormalities of breathing	R06
Pain in throat and chest	R07
Other symptoms and signs involving the circulatory and respiratory system	R09
Dizziness and giddiness	R42
Fever of other or unknown origin	R50
Headache	R51
Malaise and fatigue	R53
Syncope and collapse	R55
**Upper and lower respiratory tract infections**	
Acute nasopharyngitis	J00
Acute tonsillitis	J03
Acute upper respiratory infections of multiple and unspecified sites	J06
Influenza due to other identified influenza virus	J10
Other viral pneumonia	J128
Viral pneumonia, unspecified	J129
Pneumonia due to Streptococcus pneumoniae	J13
Bacterial pneumonia, not elsewhere classified	J15
Pneumonia due to other specified infectious organisms	J168
Pneumonia in diseases classified elsewhere	J17
Pneumonia, unspecified organism	J18
Unspecified acute lower respiratory infection	J22
Coronavirus infection, unspecified	B342
Other viral infections of unspecified site	B348
Viral infection, unspecified	B349
Coronavirus as the cause of diseases classified elsewhere	B972
Other and unspecified infectious diseases	B99
**Respiratory disorders**	
Pulmonary embolism	I26
Acute respiratory distress syndrome	J80
Pulmonary edema	J81
Pleural effusion not elsewhere classified	J90
Respiratory failure, not elsewhere classified	J96
Respiratory disorders in diseases classified elsewhere	J99
**Obstructive Airway Diseases**	
Acute bronchitis due to other specified organisms	J208
Acute bronchitis, unspecified	J209
Acute bronchiolitis due to other specified organisms	J218
Acute bronchiolitis, unspecified	J219
Other chronic obstructive pulmonary disease	J44
Asthma	J45
**Cardiac diseases**	
Viral carditis	B332
Chronic ischemic heart disease	I25
Acute pericarditis	I30
Pericarditis in diseases classified elsewhere	I32
Acute myocarditis, unspecified	I40
Myocarditis in diseases classified elsewhere	I41
Atrial fibrillation and flutter	I48
Heart failure	I50
Abnormalities of heart beat	R00
**Kidney disorders**	
Acute kidney failure	N17

Chronic kidney disease	N18
Unspecified kidney failure	N19
**Electrolyte disorders**	
Other disorders of fluid, electrolyte and acid-base balance	E87

Table S2: Result of hospitalization analysis. OR, 95% CI. Basic model controlled for age at conscription, year of conscription and children in other age groups.

Age of child in 2020	*Basic model*	*Adjusted for baseline CRF*	*Adjusted for baseline BMI, height, morbidity*	*Adjusted for parental education, income*	*Adjusted for profession*	*Full model*
			All hospitalizations March 2020 – September 2021			
N	1 551 550	1 137 763	1 551 550	1 408 588	1 551 550	1 023 722
n of cases	8 541	6 641	8 541	7 416	8 541	5 701
2-5	**0.74** (0.66-0.83)	**0.71** (0.61-0.83)	**0.76** (0.68-0.85)	**0.78** (0.70-0.88)	**0.75** (0.66-0.84)	**0.78** (0.66-0.90)
6-8	0.95 (0.86-1.05)	1.01 (0.89-1.15)	0.96 (0.87-1.07)	0.99 (0.90-1.10)	0.95 (0.86-1.05)	1.08 (0.95-1.23)
9-12	0.98 (0.90-1.06)	0.93 (0.84-1.02)	0.99 (0.92-1.08)	1.03 (0.95-1.11)	0.98 (0.91-1.07)	0.99 (0.89-1.09)
13-17	**1.16** (1.09-1.24)	**1.15** (1.07-1.24)	**1.18** (1.11-1.26)	**1.21** (1.13-1.29)	**1.16** (1.09-1.23)	**1.20** (1.10-1.29)
18-19	**1.16** (1.07-1.25)	**1.15** (1.05-1.26)	**1.17** (1.08-1.27)	**1.19** (1.09-1.29)	**1.16** (1.07-1.26)	**1.20** (1.09-1.32)
20-22	1.09 (0.99-1.18)	1.10 (1.00-1.20)	**1.10** (1.01-1.19)	**1.15** (1.05-1.25)	1.09 (0.99-1.18)	**1.15** (1.05-1.27)
22 and over	**1.16** (1.09-1.23)	**1.16** (1.09-1.24)	**1.16** (1.09-1.23)	**1.21** (1.13-1.29)	**1.15** (1.09-1.22)	**1.22** (1.13-1.31)
			Wave 1 (March-September 2020)			
n of cases	2 384	1 849	2 384	2 055	2 384	1 581
2-5	**0.72** (0.58-0.89)	**0.60** (0.44-0.82)	**0.73** (0.59-0.91)	**0.76** (0.60-0.94)	**0.72** (0.58-0.89)	**0.63** (0.46-0.88)
6-8	0.87 (0.71-1.06)	0.87 (0.67-1.13)	0.88 (0.72-1.07)	0.92 (0.75-1.12)	0.87 (0.71-1.06)	0.92 (0.70-1.20)
9-11	0.87 (0.71-1.06)	0.91 (0.75-1.10)	0.92 (0.79-1.08)	0.98 (0.84-1.16)	0.91 (0.78-1.07)	1.00 (0.82-1.22)
12-17	0.95 (0.84-1.08)	0.91 (0.78-1.06)	0.96 (0.84-1.09)	1.00 (0.88-1.15)	0.95 (0.83-1.08)	0.94 (0.80-1.10)
18-19	1.16 (0.99-1.36)	**1.23** (1.04-1.46)	1.17 (1.01-1.37)	**1.19** (1.01-1.40)	1.16 (0.99-1.35)	**1.27** (1.06-1.52)
20-22	**1.17** (1.00-1.37)	**1.24** (1.05-1.46)	1.18 (1.01-1.37)	**1.25** (1.06-1.48)	1.17 (0.99-1.36)	**1.31** (1.10-1.57)
22 and over	**1.26** (1.13-1.40)	**1.26** (1.11-1.42)	**1.26 (**1.13-1.49)	**1.35** (1.19-1.52)	**1.25** (1.12-1.39)	**1.34** (1.17-1.53)
			Wave 2 (September 2020-February 2021)			
n of cases	2 507	1 986	2 507	2 142	2 507	1 674
2-5	0.80 (0.64-1.00)	0.94 (0.71-1.24)	0.82 (0.67-1.02)	0.86 (0.68-1.08)	0.80 (0.64-1.00)	1.00 (0.75-1.33)
6-8	0.93 (0.76-1.14)	1.05 (0.82-1.34)	0.94 (0.77-1.15)	0.99 (0.81-1.22)	0.93 (0.76-1.14)	1.15 (0.90-1.48)
9-11	0.99 (0.85-1.16)	0.91 (0.75-1.09)	1.01 (0.86-1.17)	1.04 (0.88-1.22)	0.99 (0.85-1.16)	0.96 (0.80-1.17)
12-17	1.13 (0.99-1.28)	1.15 (0.99-1.33)	**1.15** (1.01-1.30)	**1.17** (1.02-1.34)	1.13 (0.99-1.28)	**1.21** (1.04-1.42)
18-19	**1.20** (1.03-1.40)	1.16 (0.97-1.37)	**1.21** (1.04-1.41)	**1.28** (1.09-1.50)	**1.20** (1.03-1.40)	**1.27** (1.07-1.52)
20-22	1.02 (0.87-1.20)	1.03 (0.87-1.24)	1.03 (0.88-1.21)	1.08 (0.91-1.28)	1.03 (0.87-1.20)	1.12 (0.93-1.35)
22 and over	**1.19** (1.07-1.32)	**1.22** (1.08-1.37)	**1.19** (1.07-1.32)	**1.29** (1.14-1.45)	**1.19** (1.07-1.32)	**1.33** (1.16-1.52)

	Wave 3 (February 2021-July 2021)					
n of cases	3 650	2 806	3 650	3 219	3 650	2 446
2-5	**0.73** (0.62-0.87)	**0.69** (0.55-0.86)	**0.75** (0.64-0.89)	**0.76** (0.64-0.90)	**0.73** (0.62-0.87)	**0.73** (0.58-0.91)
6-8	1.01 (0.88-1.17)	1.10 (0.92-1.31)	1.03 (0.89-1.19)	1.05 (0.90-1.21)	1.01 (0.88-1.17)	1.14 (0.95-1.37)
9-11	1.02 (0.91-1.14)	0.97 (0.84-1.11)	1.03 (0.92-1.16)	1.04 (0.92-1.17)	1.02 (0.91-1.14)	0.99 (0.86-1.15)
12-17	**1.33** (1.21-1.46)	**1.34** (1.20-1.50)	**1.35** (1.23-1.48)	**1.36** (1.24-1.50)	**1.33** (1.21-1.46)	**1.35** (1.21-1.52)
18-19	**1.14** (1.01-1.29)	1.11 (0.97-1.28)	**1.15** (1.02-1.30)	1.13 (0.99-1.29)	**1.14** (1.01-1.29)	1.12 (0.97-1.30)
20-22	1.09 (0.96-1.23)	1.06 (0.92-1.23)	1.10 (0.97-1.25)	1.13 (0.99-1.29)	1.08 (0.96-1.23)	1.08 (0.93-1.26)
22 and over	1.07 (0.97-1.17)	1.09 (0.98-1.21)	1.07 (0.97-1.17)	1.06 (0.96-1.18)	1.07 (0.97-1.17)	1.07 (0.95-1.20)

Table S3: Associations between children in the household and hospitalization due to COVID-19. Model controlled for age, baseline BMI, CRF, height, chronic morbidity, parental education, income, profession and place of residence in 2018. OR, 95% CI.

	Only children 2-5	*Only children 6-12*	*Only children 13-22*	*Only Children 6-22*
N	66 385	119 397	382 277	698 938
n of cases	120	426	2 504	3 690
March 2020-July 2021	**0.54** (0.42-0.71)	0.95 (0.84-1.08)	**1.28** (1.21-1.36)	**1.30** (1.22-1.39)
Wave 1	**0.49** (0.29-0.82)	**0.74** (0.57-0.98)	**1.30** (1.16-1.46)	**1.22** (1.08-1.38)
Wave 2	0.60 (0.36-1.00)	0.78 (0.59-1.02)	**1.34** (1.20-1.50)	**1.37** (1.21-1.54)
Wave 3	**0.57** (0.40-0.82)	1.18 (0.99-1.40)	**1.24** (1.13-1.36)	**1.31** (1.19-1.44)

Table S4: Result of infection analysis. OR, 95% CI.

Age of child in 2020	*Basic model*	*Adjusted for baseline CRF*	*Adjusted for baseline BMI, height, morbidity*	*Adjusted for parental education, income*	*Adjusted for profession*	*Full model*
			**All test positives**			
N	1 551 550	1 137 763	1 551 550	1 416 004	1 551 550	1 023 722
n of cases	189 270	135 363	189 270	179 085	189 270	126 845
2-5	**1.03** (1.02-1.05)	**0.97** (0.95-0.99)	**1.04** (1.01-1.05)	**0.95** (0.94-0.97)	**1.03** (1.01-1.04)	**0.91** (0.89-0.93)
6-8	**1.13** (1.11-1.15)	**1.01** (1.07-1.12)	1.24 (1.11-1.14)	**1.06** (1.04-1.07)	**1.13** (1.11-1.15)	**1.04** (1.02-1.06)
9-11	**1.25** (1.24-1.27)	**1.24** (1.19-1.24)	**1.25** (1.23-1.27)	**1.17** (1.15-1.18)	**1.25** (1.24-1.27)	**1.14** (1.12-1.16)
12-17	**1.43** (1.41-1.45)	**1.40** (1.38-1.43)	**1.42** (1.41-1.44)	**1.31** (1.29-1.33)	**1.43** (1.41-1.45)	**1.29** (1.27-1.32)
18-19	**1.34** (1.32-1.36)	**1.33** (1.30-1.35)	**1.34** (1.31-1.36)	**1.25** (1.23-1.27)	**1.34** (1.32-1.36)	**1.24** (1.22-1.27)
20-22	**1.31** (1.28-1.33)	**1.29** (1.26-1.31)	**1.30** (1.27-1.32)	**1.20** 81.18-1.22)	**1.30** (1.28-1.33)	**1.19** (1.16-1.21)
22 and over	**1.16** (1.14-1.18)	**1.20** (1.18-1.22)	**1.16 (**1.14-1.17)	**1.02** (1.00-1.03)	**1.16** (1.14-1.18)	**1.06 (**1.04-1.08)
			**Wave 1**			
n of cases	10 865	7 980	10 865	10 051	10 865	7 311
2-5	0.94 (0.87-1.01)	0.91 (0.83-1.00)	0.94 (0.87-1.01)	**0.89** (0.83-0.96)	0.94 (0.87-1.00)	**0.88** (0.80-0.98)
6-8	0.96 (0.90-1.03)	0.97 (0.88-1.06)	0.96 (0.89-1.03)	**0.92** (0.85-0.98)	0.96 (0.90-1.03)	0.93 (0.85-1.02)
9-11	1.01 (0.95-1.07)	1.00 (0.94-1.08)	1.01 (0.95-1.07)	0.96 (0.90-1.01)	1.01 (0.95-1.07)	0.96 (0.89-1.04)
12-17	**1.20** (1.14-1.26)	**1.16** (1.10-1.24)	**1.20** (1.14-1.26)	**1.13** (1.07-1.19)	**1.19** (1.13-1.26)	**1.10** (1.04-1.17)
18-19	**1.35** (1.27-1.44)	**1.38** (1.28-1.48)	**1.35** (1.26-1.44)	**1.26** (1.18-1.35)	**1.35** (1.26-1.43)	**1.30** (1.21-1.40)
20-22	**1.31** (1.22-1.40)	**1.32** (1.22-1.42)	**1.30** (1.21-1.39)	**1.23** (1.15-1.32)	**1.30** (1.21-1.39)	**1.24** (1.14-1.34)
22 and over	**1.16** (1.10-1.23)	**1.21** (1.14-1.28)	**1.15** (1.09-1.22)	**1.08** (1.02-1.15)	**1.16** (1.09-1.22)	**1.13** (1.05-1.21)
			**Wave 2**			
n of cases	81 819	58 952	81 819	77 209	81 819	55 085
2-5	1.00 (0.98-1.03)	**0.95** (0.92-0.98)	1.00 (0.98-1.03)	**0.92** (0.90-0.94)	1.00 (0.97-1.03)	**0.88** (0.85-0.91)
6-8	**1.06** (1.03-1.08)	1.02 (0.99-1.05)	**1.05** (1.03-1.08)	0.99 (0.97-1.01)	**1.06** (1.03-1.08)	**0.96** (0.93-0.99)
9-11	**1.20** (1.17-1.22)	**1.15** (1.12-1.18)	**1.20** (1.17-1.22)	**1.11** (1.10-1.14)	**1.20** (1.17 -1.22)	**1.08** (1.05-1.11)
12-17	**1.37** (1.35-1.40)	**1.35** (1.32-1.37)	**1.36** (1.34-1.39)	**1.25** (1.23-1.28)	**1.37** (1.34-1.39)	**1.24** (1.21-1.26)
18-19	**1.28** (1.25-1.31)	**1.26** (1.23-1.30)	**1.28** (1.25-1.31)	**1.20** (1.17-1.23)	**1.28** (1.25-1.31)	**1.18** (1.15-1.22)
20-22	**1.27** (1.24-1.31)	**1.26** (1.23-1.30)	**1.27** (1.23-1.30)	**1.17** (1.14-1.20)	**1.27** (1.24-1.31)	**1.16** (1.13-1.20)
22 and over	**1.17** (1.14-1.20)	**1.20** (1.17-1.23)	**1.17** (1.14-1.19)	**1.03** (1.01-1.05)	**1.17** (1.14-1.19)	**1.06** (1.03-1.09)

	Wave 3					
n of cases	96 586	68 431	96 586	91 825	96 586	64 449
2-5	**1.06** (1.04-1.08)	0.99 (0.97-1.03)	**1.06** (1.03-1.08)	0.99 (0.97-1.01)	**1.06** (1.04-1.08)	**0.94** (0.91-0.97)
6-8	**1.18** (1.16-1.21)	**1.16** (1.12-1.18)	**1.18** (1.15-1.20)	**1.12** (1.09-1.14)	**1.18** (1.16-1.21)	**1.10** (1.07-1.13)
9-11	**1.28** (1.26-1.31)	**1.25** (1.23-1.28)	**1.28** (1.26-1.31)	**1.20** (1.18-1.22)	**1.28** (1.26-1.30)	**1.18** (1.16-1.21)
12-17	**1.42** (1.40-1.45)	**1.41** (1.38-1.44)	**1.42** (1.40-1.44)	**1.32** (1.30-1.34)	**1.42** (1.40-1.45)	**1.31** (1.28-1.34)
18-19	**1.33** (1.30-1.36)	**1.31** (1.28-1.35)	**1.32** (1.29-1.35)	**1.24** (1.22-1.27)	**1.33** (1.30-1.36)	**1.24** (1.21-1.27)
20-22	**1.28** (1.25-1.31)	**1.25** (1.22-1.29)	**1.27** (1.24-1.30)	**1.18** (1.15-1.21)	**1.28** (1.25-1.31)	**1.17** (1.13-1.20)
22 and over	**1.13** (1.10-1.15)	**1.16** (1.14-1.19)	**1.12** (1.10-1.15)	0.99 (0.98-1.02)	**1.13** (1.10-1.15)	**1.04** (1.01-1.06)
