# Supplementary material for: Children in the household and risk of severe COVID-19 during the first three waves of the pandemic: a prospective registry-based cohort study of 1.5 million Swedish men
Source: BMJ Open. 2022 Aug 11;12(8):e063640. doi: 10.1136/bmjopen-2022-063640 (PMC9378946; doi:10.1136/bmjopen-2022-063640)
Supplement: Supplementary file 1 [file bmjopen-2022-063640.draft_revisions.pdf]

BMJ Open is committed to open peer review. As part of this commitment we make the peer review history of every article we publish publicly available.

When an article is published we post the peer reviewers' comments and the authors' responses online. We also post the versions of the paper that were used during peer review. These are the versions that the peer review comments apply to.

The versions of the paper that follow are the versions that were submitted during the peer review process. They are not the versions of record or the final published versions. They should not be cited or distributed as the published version of this manuscript.

BMJ Open is an open access journal and the full, final, typeset and author-corrected version of record of the manuscript is available on our site with no access controls, subscription charges or pay-per-view fees (http://bmjopen.bmj.com).

If you have any questions on BMJ Open's open peer review process please email info.bmjopen@bmj.com

Children in the household and risk of severe COVID-19: a prospective study of 1.5 million Swedish men.

Journal:	*BMJ Open*
Manuscript ID	bmjopen-2022-063640
Article Type:	Original research
Date Submitted by the Author:	06-Apr-2022

Complete List of Authors:	af Geijerstam, Agnes; University of Gothenburg Institute of Medicine, School of Public Health and Community Medicine<br>Mehlig, Kirsten; University of Gothenburg Institute of Medicine, School of Public Health and Community Medicine<br>Hunsberger, Monica; University of Gothenburg Institute of Medicine, School of Public Health and Community Medicine<br>Åberg, Maria; University of Gothenburg Institute of Medicine, School of Public Health and Community Medicine; Region Västra Götaland, Regionhälsan<br>Lissner, Lauren; University of Gothenburg Institute of Medicine, School of Public Health and Community Medicine,

Keywords:	COVID-19, Epidemiology < INFECTIOUS DISEASES, EPIDEMIOLOGY

SCHOLARONE™
Manuscripts

I, the Submitting Author has the right to grant and does grant on behalf of all authors of the Work (as defined in the below author licence), an exclusive licence and/or a non-exclusive licence for contributions from authors who are: i) UK Crown employees; ii) where BMJ has agreed a CC-BY licence shall apply, and/or iii) in accordance with the terms applicable for US Federal Government officers or employees acting as part of their official duties; on a worldwide, perpetual, irrevocable, royalty-free basis to BMJ Publishing Group Ltd ("BMJ") its licensees and where the relevant Journal is co-owned by BMJ to the co-owners of the Journal, to publish the Work in this journal and any other BMJ products and to exploit all rights, as set out in our licence.

The Submitting Author accepts and understands that any supply made under these terms is made by BMJ to the Submitting Author unless you are acting as an employee on behalf of your employer or a postgraduate student of an affiliated institution which is paying any applicable article publishing charge ("APC") for Open Access articles. Where the Submitting Author wishes to make the Work available on an Open Access basis (and intends to pay the relevant APC), the terms of reuse of such Open Access shall be governed by a Creative Commons licence – details of these licences and which Creative Commons licence will apply to this Work are set out in our licence referred to above.

Other than as permitted in any relevant BMJ Author's Self Archiving Policies, I confirm this Work has not been accepted for publication elsewhere, is not being considered for publication elsewhere and does not duplicate material already published. I confirm all authors consent to publication of this Work and authorise the granting of this licence.

For peer review only

Children in the household and risk of severe COVID-19: a prospective study of 1.5 million Swedish men.

Authors: Agnes af Geijerstam [a], Kirsten Mehlig [a], Monica Hunsberger [a], Maria Åberg [a, b], Lauren Lissner [a]

[a] School of Public Health and Community Medicine, Institute of Medicine, Sahlgrenska Academy, University of Gothenburg, Gothenburg, Sweden

[b] Region Västra Götaland, Regionhälsan, Gothenburg, Sweden

Corresponding author: Agnes af Geijerstam, School of Public Health and Community Medicine, Institute of Medicine, Sahlgrenska Academy, University of Gothenburg, P.O Box 454, 405 30 Gothenburg, Sweden. agnes.af.geijerstam@gu.se

Keywords: COVID-19, SARS-CoV-2, children, fathers, household, hospitalisation, infection, school closures, cardiorespiratory fitness, adolescence, men, population-based, cohort.

Abstract

Objective To investigate whether Swedish men living with children had elevated risk for severe COVID-19 or infection with SARS-CoV-2.

Design Prospective registry-based cohort study.

Participants 1 557 061 Swedish men, undergoing military conscription between 1968 and 2005 at a mean age of 18.3 (SD 0.73) years.

Main outcome measures Infection with SARS-CoV-2 and hospitalisation due to COVID-19 from March 2020 to September 2021.

Results There was a protective association between pre-school children at home and hospitalisation due to COVID-19 during the first and third waves compared to only older or no children at all, with odds ratios (OR) 95% confidence limits (CI) 0.64 (0.46-0.88) and 0.73 (0.58-0.91) respectively. No association was observed for living with children 6-12 years old, but for 13-17 years old the risk increased. Age in 2020 did not explain these associations. Further adjustment for socioeconomic and health factors did not attenuate the results. Exposure to pre-school children also had a protective association with testing positive with SARS-CoV-2, with or without hospitalisation, OR = 0.91 (95% CI 0.89-0.93), while living with children of other ages was associated with increased odds of infection.

Conclusions Cohabiting with pre-school children was associated with reduced risk for severe COVID-19. Living with school-age children between 6 and 12 years had no association with severe COVID-19, but sharing the household with teenagers and young adults was associated with elevated risk. Our results

For peer review only

are of special interest since pre-schools and compulsory schools (age 6-15) in Sweden were not closed in 2020.

Strengths and limitations to this study

As schools were not closed in Sweden, the effects of living with children in this country constitutes an important comparison point.

Our large study population, based on validated registry data and including key covariates reflecting the conscript's comorbidities and physical condition, is an important strength.

The major limitation is that only men were included.

Due to the observational design, we are also unable to rule out that unknown factors influenced the results.

For peer review only

Background

Early evidence showed that children were less affected by the SARS-CoV-2 virus than adults and adolescents(1,2). This notwithstanding, with the initial attempts to curb transmission by school closures and lockdowns, children around the world saw their lives changed. The effects of school closures on the spread of infectious disease had been discussed before the COVID-19 pandemic. Modelling studies focusing on influenza showed the effectiveness varied depending on the basic reproduction number and on whether children were driving the attack rates due to less immunity compared to adults(3–5). Despite the conflicting evidence on the effectiveness of school closures in relation to the character of SARS-CoV-2, it was widely implemented as a non-pharmaceutical intervention (NPI).

Sweden was an exception where compulsory schools (ages 6-15) and pre-schools were kept open. Attendance was mandatory and enforced. Schools and pre-schools were to implement preventive measures such as distancing and hand hygiene, and avoid unnecessary mixing of classes and teachers(6), while teaching in upper secondary school was moved online.

Two large studies have been published on the risk of parental infection posed by living with children. Wood et al with a population of over 300 000 health care workers and the adults they live with in Scotland(7), and Forbes et al with the COVID-19: OpenSAFELY cohort of 12 million adults in England(8). Neither could show an increased risk related to living with children of any age during wave 1. During wave 2 there was a small absolute risk associated with children of any age living at home, except for younger children as reported in the Scottish cohort. Part of the risk increase was attributed to the return to schools and preschools in September 2020.

Men are disproportionally affected by COVID-19, comprising 74% of those admitted to intensive care in Sweden(9). In the present study, we had the opportunity to examine a large part of the male population in Sweden, for whom information is available on early health factors that influence severity of COVID-19(e12,13). As schools and preschools were open in Sweden, it is a unique starting point for investigating the effects of sharing a household with children during the pandemic.

Methods

Study design

This is a prospective cohort study based on data from the Swedish Military Conscription Registry, combined with a socioeconomic population registry (LISA) from Statistics Sweden as well as the Swedish national hospital and intensive care registries.

Population studied

The Swedish military conscript registry contains information about 1 949 891 Swedish individuals who enlisted for military service between late 1968 and 2005. During those years Swedish law required all male

For peer review only

citizens to enlist, except for those in prison or those with severe chronic somatic or psychiatric conditions or functional disabilities (approximately 2-3% annually).

Patient and public involvement statement

As this is a registry-based study, there have been no patient or public involvement.

Main independent variables

Children at home

Data about children registered at the same address as the men in the cohort was retrieved from Statistics Sweden. Most of the men are assumed to be fathers, biological or not, but they could also be grandfathers or lodgers. The age brackets correspond to Swedish pre-school, primary school, middle school, and lower and upper secondary school. No children in the youngest bracket would have started school during 2020-2021 but those aged 6 in 2020 would have been in pre-school during wave 1 and then in first grade during wave 2 and 3. School in Sweden is compulsory from 6 to 15 years of age and about 85% of all 16–18-year-olds attend upper secondary school. About 90% of all Swedish 2-year-olds attend pre-school, with even higher rates for 4-5 year-olds(12).

Confounding variables

Weight and height were measured by standard anthropometric measurement techniques, and continuous BMI values (kg/m^2) were calculated, as BMI has been showed to be one of the major risk factors for severe COVID-19. Earlier studies on the same cohort have shown an association between BMI and cardiorespiratory fitness (CRF) in early adulthood and later risk of severe disease (11,13–15). There is also a known correlation between CRF, height and BMI at conscription and the probability of having children (16).

Morbidity at baseline

All medical diagnoses prior to conscription are recorded in the conscript registry. Illness that could have affected the ability or decision to have children, as well as later risk of severe COVID-19 was controlled for using ICD-codes (International Classification of Diseases) for respiratory disease, cardiovascular disease, diabetes, kidney disease and malignant cancers (16,17).

Socioeconomic indicators

Parental education was considered a proxy for socioeconomic position of the household. Using data from the LISA registry, it was based on the highest in the household and categorized as: low (up to 9 years), medium (upper secondary school diploma with ≤2 years at university) and high education (≥3 years at university). Data on home municipality was collapsed into three categories – large, medium and small towns and the municipalities nearby(18). Disposable family income was categorized into low, medium, and high income based on tertiles.

For peer review only

From LISA we also had information on profession. High risk occupation was defined a posteriori as those where the risk of hospitalisation due to COVID-19 was 0.28% and higher. This included health care personnel, bus drivers, restaurant workers, service personnel, industrial workers, social workers and primary school teachers (N=314 834).

Analytic sample

Originally comprising 1 949 891 conscripts, the cohort was reduced to 1 559 187 men after exclusions (Figure 1). 1 557 061 had information in LISA on children at home. Those who died during 2020 and until February 2021 (n=5 012) were censored in the analysis prior to each wave, giving an at risk population of 1 555 835 at the beginning of wave 1, 1 552 040 at wave 2 and 1 549 514 at wave 3. Characteristics of the population, together with crude outcome data, are shown in Table 1.

Figure 1 here

Table 1: Characteristics of the study population.

Conscription year	*1968-1975*	*1976-1985*	*1986-1995*	*1996-2005*	*All decades*
N in 2020	282 828	440 991	495 775	337 467	1 557 061
Age at conscription, mean (SD)	18.5 (0.65)	18.3 (0.82)	18.3 (0.76)	18.2 (0.58)	18.3 (0.73)
Age in 2020 mean (SD)	65.9 (1.94)	57.5 (2.98)	48.0 (3.00)	38.3 (2.91)	51.8 (9.89)
	Children at home (age in 2018)				
Child of any age	43 220 (15.3)	177 630 (40.3)	333 582 (67.3)	212 025 (62.8)	790 604 (50.8)
0-3 years (%)	292 (0.1)	3 151 (0.7)	38 873 (7.9)	111 926 (33.2)	154 242 (9.9)
4-6 years (%)	457 (0.2)	5 657 (1.3)	62 168 (12.5)	92 332 (27.4)	160 612 (10.3)
7-10 years (%)	1 275 (0.5)	16 576 (3.8)	124 800 (25.2)	79 418 (23.5)	222 069 (14.3)
11-15 years (%)	3 967 (1.4)	47 911 (10.9)	168 507 (34)	35 318 (10.5)	255 703 (16.4)
16-17 years (%)	3 597 (1.3)	35 946 (8.2)	68 234 (13.8)	5 549 (1.6)	113 326 (7.3)
18-20 years (%)	4 719 (1.7)	40 733 (9.2)	50 320 (10.2)	2 641 (0.8)	98 413 (6.3)
20 and older (%)	34 259 (12.1)	94 456 (21.4)	51 782 (10.4)	15 983 (4.7)	196 480 (12.6)
	Hospitalisations due to COVID-19				
N, March 2020-Sep 2021 (%)	2 261 (0.80)	3 179 (0.72)	2 455 (0.50)	760 (0.23)	8 655 (0.56)
With children at home (%)	426 (18.8)	1 318 (41.5)	1 616 (65.8)	463 (61)	3 823 (44.2)
	PCR confirmed infection with SARS-CoV-2				
N, March 2020-Sep 2021(%)	18 471 (6.5)	50 272 (11.4)	72 878 (14.7)	47 825 (14.2)	189 446 (12.2)
With children at home (%)	3 793 (20.5)	24 279 (48.3)	54 920 (75.4)	32 671 (68.3)	115 663 (61.1)

Outcome variables

Hospitalisation due to Covid-19

Using the Swedish personal identification number, the full sample was linked to the National Patient Register and the Swedish Intensive Care Registry. From these, all cases between March 2020 and September 2021 with a main diagnosis of ICD U071 for test verified infection with SARS-CoV-2 and U072 for clinically diagnosed COVID-19 were identified. Records with U071 or U072 as a secondary diagnosis were counted as cases if the main diagnosis was clinically related to COVID-19 (table S1). All hospital-care requiring illness was considered severe COVID-19. Register data based on Swedish hospital records have high validity (19).

Infection with COVID-19

Free PCR-testing began in summer 2020. Previously, testing was mainly done in hospitals. Therefore, data from this period is limited and not representative of the actual infection rates, as is seen in the comparison between testing and hospitalisations in Figure 2. All positive tests were to be registered in the Sminet registry according to the Swedish Communicable Diseases Act. Data from Sminet was extracted on September 21st, 2021 and covers all positives until that day.

Figure 2 here

Statistical analysis

The main independent variable was analysed as a binary variable in each age interval. Logistic regression was used to calculate the odds for hospitalisation and infection due to COVID-19 by this exposure category, adjusted for children in other age brackets, place of residence, income and profession in 2018, linear, quadratic, and cubic terms of BMI, as well as CRF, height, parental education and chronic disease at conscription. All regression models were adjusted for exact age at conscription and year of conscription examination, and thus indirectly for age in 2020. Because overall events of hospitalisation were rare, we used penalized likelihood estimation (Firth method) to reduce (potential) small-sample bias in maximum likelihood estimation

Statistical analyses were performed with SAS version 9.4 (SAS Institute, Cary, NC). Statistical significance was set at 0.05 (2-sided tests).

Ethics approval statement

The study conforms to the principles outlined in the Declaration of Helsinki. The Ethics Committee of the University of Gothenburg and Confidentiality Clearance at Statistics Sweden approved the study (EPN Reference numbers EPN 462-14 and 567-15; T174-15, T653-17, T196-17, T2019-05875, T 2020-01325, T2020-02420, T2021-00797, T2021-03310). The requirement for informed consent was waived by

For peer review only

the Ethics Committee of the University of Gothenburg for secondary analysis of existing data. The data was pseudonymized before being accessed by the study authors.

Results

Hospitalisations

Having a child of preschool age at home had a protective association with hospitalisation due to COVID-19 during the pandemic from March 2020 to July 2021 (Figure 3). This association is statistically significant in the first and third wave with OR=0.64 (95% CI 0.46-0.88) and 0.73 (0.58-0.91). During the second wave, no association could be seen (OR=1.06 (0.80-1.41)). In contrast, no associations between living with children of primary school age and hospitalisation were observed. Sharing the household with children 13 years of age or older conveyed an overall excess risk of hospitalisation.

Figure 3 here

None of the results was attenuated after adjustment for covariates (table S2). Exposures describing children of different age groups were not mutually exclusive, as many fathers live with more than one child. For the combined waves, the protective association between the youngest children and hospitalisation due to COVID-19 was strengthened to OR 0.54 (95% CI 0.42-0.71) when we considered a separate category of those with only pre-school aged cohabitants (table S3).

Infection

Living in a household with children over 5 was associated with an increased risk for infection with SARS-CoV-2. The same pattern can be seen for all waves, with odds ratios significantly higher for all age groups apart from the pre-school children who had a significant protective association (Figure 4) with OR=0.91 (95% CI 0.89-0.93). Wave specific results can be found in Supplementary table S4.

Figure 4 here

Adjustment for high-risk profession in the main analysis did not attenuate the effect estimates. To evaluate the effects of isolating with children not yet in compulsory school we examined the subset of men in high-risk occupations only: having younger children still gave a protective association with OR= 0.94 (95% CI 0.91-0.98). Again, when looking at those living with pre-school children only, the association was stronger with OR=0.73 (95% CI 0.69-0.77).

Discussion

For peer review only

In this paper we demonstrate a robust protective association between residing with children of pre-school age and the risk of severe COVID-19 during two of the three waves of the pandemic in Sweden.

The comparable studies from Scotland and England showed that having children at home (any age in England, 0-11 in Scotland) was not associated with increased risk of infection or severe disease in spring 2020. During the second wave risk of infection and COVID-19 related hospital admission was increased in the OpenSAFELY cohort but not in the Scottish cohort(7,8). This finding was attributed in part to the school closures during spring and the reopening after summer. Our results fit the same pattern but cannot be explained the same way, since the schools were open. An earlier Swedish study presents a similar small increase in infections among parents of lower-secondary school age children during the first wave(20).

High-risk profession did not affect the associations. With our smaller age brackets, we were able to single out associations in parents of children in pre-school, which were distinctly different from those with children of older ages and highlights a methodological strength of our study.

No national figures of school or pre-school attendance are available, but in the numbers of the official statistics bureau of Gothenburg (pop. 583 056) attendance was distinctly lower in March 2020, and sick leave was higher during the pandemic compared to 2019. The possibility to self-isolate with younger children could of course contribute to the protective association. The Swedish pre-school teacher's union reported a large drop in attendance in March 2020. After March though, the majority returned to pre-school (21).

Our large study population, based on validated registry data and including key covariates reflecting the conscript's comorbidities and physical condition, is an important strength. However, since this is an observational study, we cannot rule out that unknown factors such as behavioural differences between those living with or without children of different ages influenced the results. The major limitation is that only men were included in this study. Furthermore, all information from the LISA registry is from 2018. However, only minor changes in these covariates can be expected during 2019-2020, except for the oldest children who might have left home during this period.

We were not able to include the effects of vaccination, but as the main part of our cohort were vaccinated from May 2021, the potential effect is restricted to the tapering off of wave 3, with very few cases overall.

Clinical and public health implications

The decision to keep mandatory schools open in Sweden constitute a comparison point. A recent review of studies trying to evaluate the effect of school closures on community transmission concluded that "The true independent effect of school closures from the first wave around the world may simply be unknowable"(22). The model calculations included in the review all had problems differentiating between NPI's implemented simultaneously. Closing schools was meant to control community transmission by

For peer review only

limiting transmission between children and subsequently between children and parents. As our study shows, the odds ratio for infection was higher for men living with children of all age groups except those aged 2-5. This could be expected due to the greater number of contacts in school but is still comparable to transmission patterns where schools were closed during spring 2020.

The finding that living with children aged 2-5 was associated with lower risk of severe COVID-19 does raise questions. If the effect is not entirely due to behavioural differences or parental health it could be speculated that simultaneous infection with other respiratory viruses commonly occurring in this group, such as rhinovirus, could be protective, as has been shown in vitro(23,24). Both wave 1 and wave 3 coincide with the months when the Swedish Social Insurance Agency normally distributes the most compensation for care of sick child (mainly due to cold viruses in early spring).

Conclusion

Young children seem to have played a minor part in the community transmission of COVID-19, even though pre-schools remained open in Sweden. As this study shows, this age group conveyed a protective association both with the risk of infection and with the risk of severe sickness in adult men living in the same household. Having children between 6 and 12 years in the household was associated with a small increase in odds of infection, but not with severe disease. Having teenagers in the household was associated with increased rates of infection as well as severe disease in their fathers. These associations are similar in magnitude to those reported in other settings where schools were closed.

Legends

Figure 1: Creation of analytical sample.

Table 1: Characteristics of the study population.

Figure 2: Weekly admissions to hospital due to COVID-19 in Sweden and weekly registered PCR-verified infections between March 2020 and September 2020. Note that tests were not widely available until July 2020.

Figure 3: Associations between children in the household and hospitalisation due to COVID-19 (n=1 557 061). Odds ratios with 95% CI. Model controlled for children in other age groups, age, baseline BMI, CRF, height, chronic morbidity, parental education, income, profession and place of residence in 2018.

Figure 4: Associations between children in the household and testing positive for SARS-CoV-2 March 2020-September 2021 (n=1 557 061). Odds ratios with 95% CI. Model controlled for conscript's age, BMI, CRF, height, chronic morbidity, parental education and place of residence in 2018.

For peer review only

Statements

Contributorship statement: AG, LL and MÅ initiated the project. AG and KM performed all statistical analyses. AG had main responsibility for writing the article. All authors contributed to the structure and content of the manuscript and have read and approved of the final draft. LL and MÅ share last authorship.

Competing interest statement: All authors have completed the Unified Competing Interest form (available on request from the corresponding author) and declare: no support from any other than above mentioned organization for the submitted work; no financial relationships with any organizations that might have an interest in the submitted work in the previous three years, no other relationships or activities that could appear to have influenced the submitted work.

Funding: This work was supported by the EpiLife-Teens Research Program (FORMAS-2012-00038), by the Swedish ALF-agreement (ALFGBG-720201 and ALFGBG-813511), the Swedish Research Council (02508, VRREG 2019-00193, 2020-05792) and Swedish Research Council for Health, Working Life and Welfare (2021-00304). The funding sources had no role in study design, collection, analysis and interpretation of data, the writing of the report, or the decision to submit the article for publication.

Data sharing statement: The data used in this study is available upon request from The Swedish Defence Conscription and Assessment Agency and the National Board of Health and Welfare.

Transparency statement: AG affirms that the manuscript is an honest, accurate, and transparent account of the study being reported and that no important aspects of the study have been omitted.

Dissemination to participants and related patient and public communities: We will make the findings of this article public with a press release and via media contacts.

For peer review only

References

1. Viner RM, Mytton OT, Bonell C, Melendez-Torres G, Ward J, Hudson L, et al. Susceptibility to SARS-CoV-2 infection among children and adolescents compared with adults: a systematic review and meta-analysis. JAMA pediatrics. 2021;175(2):143–56.

2. Ludvigsson JF. Systematic review of COVID-19 in children shows milder cases and a better prognosis than adults. Acta paediatrica. 2020;109(6):1088–95.

3. Cauchemez S, Ferguson NM, Wachtel C, Tegnell A, Saour G, Duncan B, et al. Closure of schools during an influenza pandemic. The Lancet infectious diseases. 2009;9(8):473–81.

4. Mangtani P. Impact of school closures on an influenza pandemic: scientific evidence base review. 2014;

5. Jackson C, Mangtani P, Hawker J, Olowokure B, Vynnycky E. The effects of school closures on influenza outbreaks and pandemics: systematic review of simulation studies. PloS one. 2014;9(5):e97297.

6. Utbildningsdepartementet. Förordning (2020:115) om utbildning på skolområdet och annan pedagogisk verksamhet vid spridning av viss smitta [Internet]. Mar 13, 2020. Available from: https://www.riksdagen.se/sv/dokument-lagar/dokument/svensk-forfattningssamling/forordning-2020115-om-utbildning-i-vissa_sfs-2020-115

7. Wood R, Thomson E, Galbraith R, Gribben C, Caldwell D, Bishop J, et al. Sharing a household with children and risk of COVID-19: a study of over 300 000 adults living in healthcare worker households in Scotland. Archives of disease in childhood. 2021;

8. Forbes H, Morton CE, Bacon S, McDonald HI, Minassian C, Brown JP, et al. Association between living with children and outcomes from covid-19: OpenSAFELY cohort study of 12 million adults in England. bmj. 2021;372.

9. Public Health Agency of Sweden. Bekräftade fall i Sverige – daglig uppdatering [Internet]. Public Health Agency of Sweden; Available from: https://www.folkhalsomyndigheten.se/smittskydd-beredskap/utbrott/aktuella-utbrott/covid-19/statistik-och-analyser/bekraftade-fall-i-sverige/

10. Af Geijerstam A, Mehlig K, Börjesson M, Robertson J, Nyberg J, Adiels M, et al. Fitness, strength and severity of COVID-19: a prospective register study of 1 559 187 Swedish conscripts. BMJ open. 2021;11(7):e051316.

11. Robertson J, Adiels M, Lissner L, Mehlig K, Af Geijerstam A, Lindgren M, et al. BMI in early adulthood is associated with severe COVID-19 later in life–a prospective cohort study of 1.5 million Swedish men. Obesity. 2022;

12. Förskola – Barn och grupper – Riksnivå [Internet]. Available from: https://www.skolverket.se/skolutveckling/statistik

13. Gao M, Piernas C, Astbury NM, Hippisley-Cox J, O'Rahilly S, Aveyard P, et al. Associations between body-mass index and COVID-19 severity in 6· 9 million people in England: a prospective, community-based, cohort study. The Lancet Diabetes & Endocrinology. 2021;

For peer review only

14. Hamer M, Gale CR, Kivimäki M, Batty GD. Overweight, obesity, and risk of hospitalization for COVID-19: A community-based cohort study of adults in the United Kingdom. Proceedings of the National Academy of Sciences. 2020;117(35):21011–3.

15. Clark A, Jit M, Warren-Gash C, Guthrie B, Wang HH, Mercer SW, et al. Global, regional, and national estimates of the population at increased risk of severe COVID-19 due to underlying health conditions in 2020: a modelling study. The Lancet Global Health. 2020;8(8):e1003–17.

16. Barclay K, Kolk M. The influence of health in early adulthood on male fertility. Population and Development Review. 2020;46(4):757–85.

17. Glazer CH, Bonde JP, Eisenberg ML, Giwercman A, Hærvig KK, Rimborg S, et al. Male infertility and risk of nonmalignant chronic diseases: a systematic review of the epidemiological evidence. In Thieme Medical Publishers; 2017. p. 282–90.

18. Sveriges kommuner och regioner. Kommungruppsindelning [Internet]. Available from: https://skr.se/skr/tjanster/kommunerochregioner/faktakommunerochregioner/kommungruppsindelning.2051.html

19. Ludvigsson JF, Andersson E, Ekbom A, Feychting M, Kim J-L, Reuterwall C, et al. External review and validation of the Swedish national inpatient register. BMC public health. 2011;11(1):1–16.

20. Vlachos J, Hertegård E, Svaleryd HB. The effects of school closures on SARS-CoV-2 among parents and teachers. Proceedings of the National Academy of Sciences. 2021;118(9).

21. Fock C. Coronatapp: 9 av 10 förskolebarn hölls hemma. Förskolan [Internet]. 2020 Jul 6; Available from: https://www.lararen.se/forskolan/coronaviruset/coronatapp-9-av-10-forskolebarn-holls-hemma

22. Walsh S, Chowdhury A, Braithwaite V, Russell S, Birch JM, Ward JL, et al. Do school closures and school reopenings affect community transmission of COVID-19? A systematic review of observational studies. BMJ Open. 2021 Aug 1;11(8):e053371.

23. Dee K, Goldfarb DM, Haney J, Amat JA, Herder V, Stewart M, et al. Human rhinovirus infection blocks SARS-CoV-2 replication within the respiratory epithelium: implications for COVID-19 epidemiology. Journal of Infectious Diseases. 2021;

24. Kuitunen I, Artama M, Haapanen M, Renko M. Rhinovirus spread in children during the COVID-19 pandemic despite social restrictions—A nationwide register study in Finland. Journal of Medical Virology. 2021;93(10):6063–7.

For peer review only

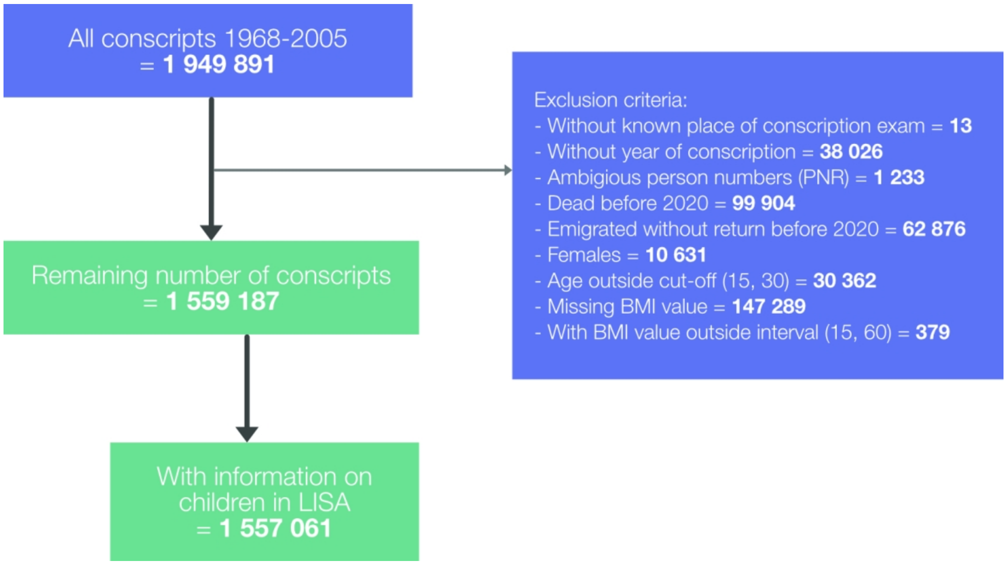

136x76mm (300 x 300 DPI)

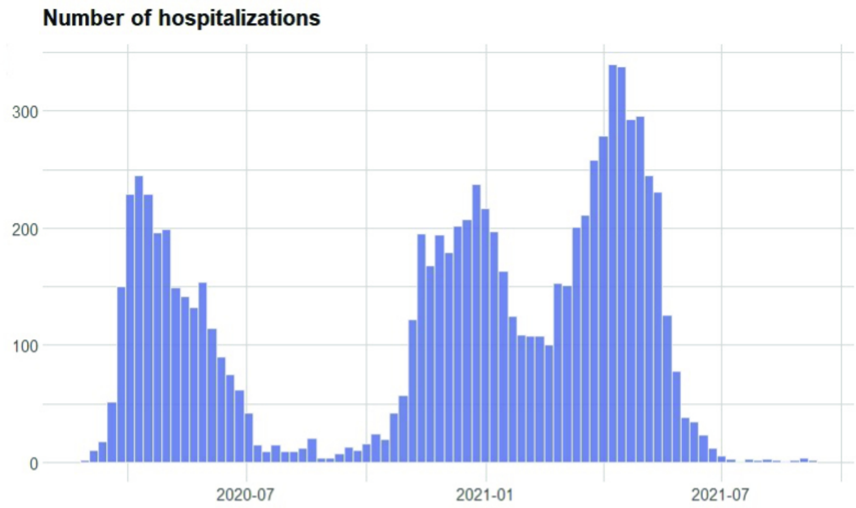

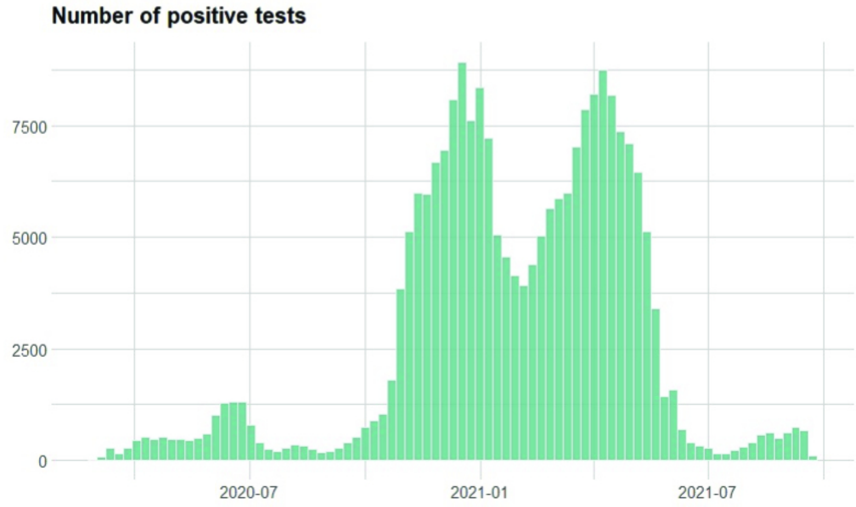

72x92mm (300 x 300 DPI)

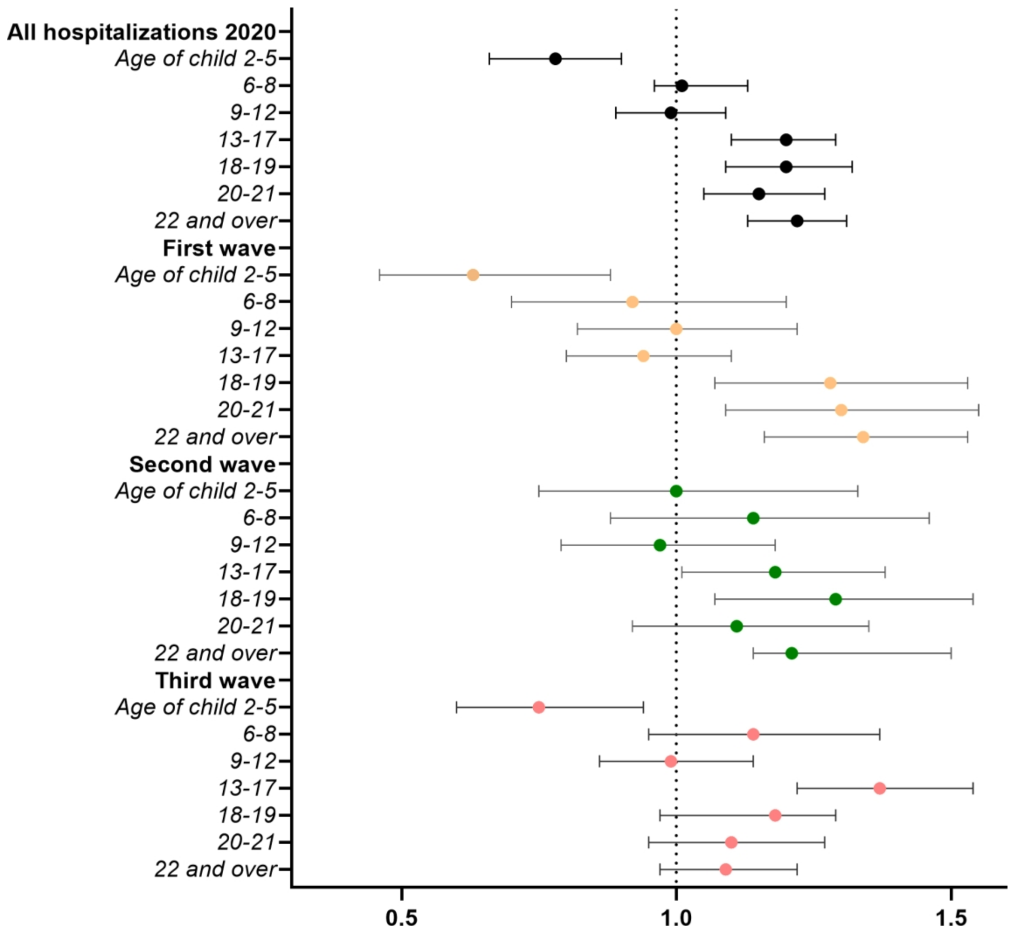

166x152mm (300 x 300 DPI)

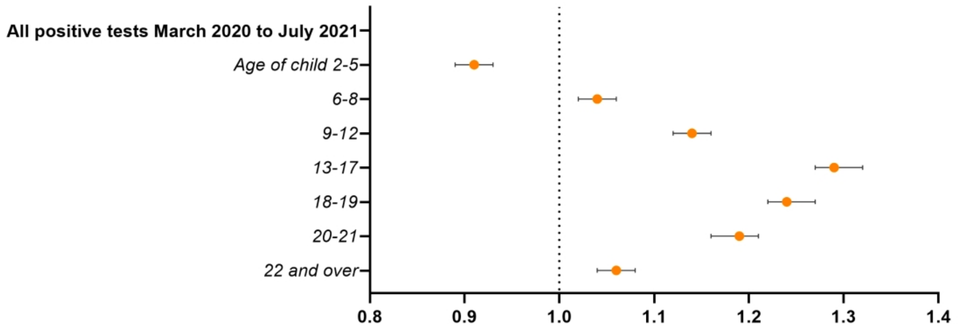

215x79mm (300 x 300 DPI)

Table S1. Included main diagnoses divided by categories when COVID-19 is secondary diagnosis.

Included categories	ICD-codes
**COVID-related symptoms**	
Cough	R05
Abnormalities of breathing	R06
Pain in throat and chest	R07
Other symptoms and signs involving the circulatory and respiratory system	R09
Dizziness and giddiness	R42
Fever of other or unknown origin	R50
Headache	R51
Malaise and fatigue	R53
Syncope and collapse	R55
**Upper and lower respiratory tract infections**	
Acute nasopharyngitis	J00
Acute tonsillitis	J03
Acute upper respiratory infections of multiple and unspecified sites	J06
Influenza due to other identified influenza virus	J10
Other viral pneumonia	J128
Viral pneumonia, unspecified	J129
Pneumonia due to Streptococcus pneumoniae	J13
Bacterial pneumonia, not elsewhere classified	J15
Pneumonia due to other specified infectious organisms	J168
Pneumonia in diseases classified elsewhere	J17
Pneumonia, unspecified organism	J18
Unspecified acute lower respiratory infection	J22
Coronavirus infection, unspecified	B342
Other viral infections of unspecified site	B348
Viral infection, unspecified	B349
Coronavirus as the cause of diseases classified elsewhere	B972
Other and unspecified infectious diseases	B99
**Respiratory disorders**	
Pulmonary embolism	I26
Acute respiratory distress syndrome	J80
Pulmonary edema	J81
Pleural effusion not elsewhere classified	J90
Respiratory failure, not elsewhere classified	J96
Respiratory disorders in diseases classified elsewhere	J99
**Obstructive Airway Diseases**	
Acute bronchitis due to other specified organisms	J208
Acute bronchitis, unspecified	J209
Acute bronchiolitis due to other specified organisms	J218
Acute bronchiolitis, unspecified	J219
Other chronic obstructive pulmonary disease	J44
Asthma	J45
**Cardiac diseases**	
Viral carditis	B332
Chronic ischemic heart disease	I25
Acute pericarditis	I30
Pericarditis in diseases classified elsewhere	I32
Acute myocarditis, unspecified	I40
Myocarditis in diseases classified elsewhere	I41
Atrial fibrillation and flutter	I48
Heart failure	I50
Abnormalities of heart beat	R00

For peer review only

**Kidney disorders**	
Acute kidney failure	N17
Chronic kidney disease	N18
Unspecified kidney failure	N19
**Electrolyte disorders**	
Other disorders of fluid, electrolyte and acid-base balance	E87

For peer review only

Table S2: Result of hospitalization analysis. OR, 95% CI. Basic model includes adjusting for age in 2020 and children of other ages. Reference for each age bracket is children in other brackets or no children at all.

Age of child in 2020	*Basic model*	*Adjusted for baseline CRF*	*Adjusted for baseline BMI, height, morbidity*	*Adjusted for parental education, income, region*	*Adjusted for profession*	*Full model*
	All hospitalizations March 2020 – September 2021					
N	1 557 061	1 142 222	1 557 061	1 420 206	1 557 061	1 027 123
n of cases	8 655	6 731	8 655	7 497	8 655	5 765
2-5	**0.74** (0.66-0.83)	**0.72** (0.62-0.84)	**0.76** (0.68-0.85)	**0.78** (0.70-0.88)	**0.75** (0.66-0.84)	**0.78** (0.66-0.90)
6-8	0.95 (0.86-1.05)	1.02 (0.89-1.16)	0.96 (0.87-1.06)	0.99 (0.90-1.10)	0.95 (0.86-1.05)	1.01 (0.95-1.13)
9-12	0.98 (0.90-1.06)	0.94 (0.84-1.02)	0.99 (0.92-1.08)	1.03 (0.95-1.11)	0.98 (0.91-1.06)	0.99 (0.89-1.10)
13-17	**1.16** (1.09-1.24)	**1.16** (1.07-1.25)	**1.18** (1.10-1.26)	**1.21** (1.13-1.29)	**1.16** (1.08-1.23)	**1.19** (1.10-1.29)
18-19	**1.16** (1.07-1.25)	**1.16** (1.06-1.27)	**1.17** (1.08-1.27)	**1.18** (1.09-1.29)	**1.16** (1.07-1.26)	**1.20** (1.09-1.32)
20-22	1.09 (0.99-1.18)	1.10 (0.99-1.20)	**1.10** (1.01-1.19)	**1.14** (1.05-1.25)	1.08 (0.99-1.17)	**1.15** (1.04-1.27)
22 and over	**1.15** (1.08-1.21)	**1.17** (1.09-1.24)	**1.15** (1.08-1.21)	**1.20** (1.12-1.28)	**1.14** (1.08-1.21)	**1.20** (1.13-1.30)
	Wave 1 (March-September 2020)					
n of cases	2 430	1 884	2 430	2 096	2 430	1 612
2-5	**0.71** (0.57-0.89)	**0.60** (0.44-0.82)	**0.73** (0.58-0.91)	**0.75** (0.60-0.94)	**0.72** (0.57-0.89)	**0.64** (0.46-0.88)
6-8	0.86 (0.70-1.05)	0.86 (0.66-1.12)	0.87 (0.72-1.06)	0.91 (0.74-1.12)	0.87 (0.71-1.06)	0.91 (0.70-1.19)
9-11	0.91 (0.78-1.07)	0.91 (0.75-1.10)	0.93 (0.79-1.08)	0.99 (0.84-1.16)	0.91 (0.78-1.07)	1.00 (0.82-1.22)
12-17	0.94 (0.83-1.07)	0.90 (0.78-1.05)	0.96 (0.84-1.09)	1.00 (0.87-1.14)	0.94 (0.83-1.07)	0.94 (0.80-1.10)
18-19	1.16 (0.99-1.35)	**1.23** (1.04-1.46)	1.17 (1.00-1.37)	**1.18** (1.00-1.41)	1.15 (0.99-1.35)	**1.26** (1.05-1.51)
20-22	1.16 (0.99-1.35)	**1.22** (1.03-1.45)	1.17 (1.00-1.37)	**1.24** (1.05-1.46)	1.15 (0.99-1.34)	**1.30** (1.09-1.55)
22 and over	**1.24** (1.11-1.38)	**1.24** (1.10-1.40)	**1.24 (**1.12-1.38)	**1.33** (1.18-1.51)	**1.24** (1.11-1.38)	**1.32** (1.15-1.51)
	Wave 2 (September 2020-February 2021)					
n of cases	2 575	2 041	2 575	2 182	2 575	1 707
2-5	**0.80** (0.64-0.99)	0.94 (0.72-1.24)	0.82 (0.66-1.03)	0.86 (0.68-1.08)	0.80 (0.64-1.00)	1.06 (0.80-1.41)
6-8	0.93 (0.76-1.12)	1.04 (0.81-1.33)	0.94 (0.77-1.15)	0.99 (0.80-1.21)	0.93 (0.76-1.13)	1.15 (0.90-1.48)
9-11	0.99 (0.85-1.16)	0.90 (0.75-1.11)	1.00 (0.86-1.17)	1.04 (0.88-1.22)	0.99 (0.85-1.16)	0.96 (0.78-1.17)
12-17	1.11 (0.97-1.26)	1.15 (0.99-1.32)	1.14 (1.00-1.29)	**1.17** (1.02-1.33)	1.12 (0.99-1.26)	**1.22** (1.04-1.41)
18-19	**1.20** (1.03-1.40)	1.16 (0.98-1.38)	**1.21** (1.04-1.41)	**1.28** (1.09-1.50)	**1.20** (1.03-1.40)	**1.28** (1.07-1.52)
20-22	1.01 (0.86-1.18)	1.02 (0.85-1.21)	1.02 (0.87-1.20)	1.08 (0.91-1.28)	1.01 (0.86-1.18)	1.11 (0.92-1.35)

For peer review only

22 and over	**1.17** (1.05-1.31)	**1.19** (1.07-1.35)	**1.17** (1.05-1.30)	**1.27** (1.12-1.42)	**1.16** (1.05-1.29)	**1.31** (1.15-1.50)
	Wave 3 (February 2021-July 2021)					
n of cases	3 650	2 806	3 650	3 219	3 650	2 446
2-5	**0.73** (0.62-0.87)	**0.69** (0.55-0.86)	**0.75** (0.64-0.89)	**0.76** (0.64-0.90)	**0.73** (0.62-0.87)	**0.73** (0.58-0.91)
6-8	1.01 (0.88-1.17)	1.10 (0.92-1.31)	1.03 (0.90-1.19)	1.05 (0.90-1.21)	1.01 (0.88-1.17)	1.14 (0.95-1.37)
9-11	1.02 (0.91-1.14)	0.97 (0.84-1.11)	1.04 (0.93-1.16)	1.04 (0.92-1.17)	1.02 (0.91-1.14)	0.99 (0.86-1.14)
12-17	**1.33** (1.21-1.46)	**1.34** (1.20-1.50)	**1.35** (1.23-1.48)	**1.36** (1.23-1.50)	**1.33** (1.21-1.46)	**1.35** (1.21-1.52)
18-19	**1.14** (1.01-1.28)	1.11 (0.97-1.27)	**1.15** (1.02-1.30)	1.13 (1.00-1.29)	**1.14** (1.01-1.29)	1.12 (0.97-1.30)
20-22	1.08 (0.96-1.23)	1.06 (0.92-1.20)	1.10 (0.97-1.24)	1.13 (0.99-1.29)	1.08 (0.96-1.23)	1.08 (0.93-1.26)
22 and over	1.07 (0.97-1.17)	1.09 (0.98-1.21)	1.07 (0.97-1.17)	1.07 (0.96-1.18)	1.07 (0.99-1.16)	1.07 (0.95-1.20)

Table S3: Associations between children in the household and hospitalization due to COVID-19. Model controlled for age, baseline BMI, CRF, height, chronic morbidity, parental education, income, profession and place of residence in 2018. OR, 95% CI.

	Only children 2-5	***Only children 6-12***	***Only children 13-22***	***Only Children 6-22***
N	66 385	119 397	382 277	698 938
n of cases	120	426	2 504	3 690
March 2020-July 2021	**0.54** (0.42-0.71)	0.95 (0.84-1.08)	**1.28** (1.21-1.36)	**1.30** (1.22-1.39)
Wave 1	**0.49** (0.29-0.82)	**0.74** (0.57-0.98)	**1.30** (1.16-1.46)	**1.22** (1.08-1.38)
Wave 2	0.60 (0.36-1.00)	0.78 (0.59-1.02)	**1.34** (1.20-1.50)	**1.37** (1.21-1.54)
Wave 3	**0.57** (0.40-0.82)	1.18 (0.99-1.40)	**1.24** (1.13-1.36)	**1.31** (1.19-1.44)

For peer review only

Table S4: Result of infection analysis. OR, 95% CI. Basic model includes adjusting for age in 2020 and children of other ages. Reference for each age bracket is children in other brackets or no children at all.

Age of child in 2020	*Basic model*	*Adjusted for baseline CRF*	*Adjusted for baseline BMI, height, morbidity*	*Adjusted for parental education, income*	*Adjusted for profession*	*Full model*
	**All test positives**					
N	1 557 061	1 142 222	1 557 061	1 420 206	1 557 061	1 027 123
n of cases	189 446	135 503	189 446	179 211	189 446	126 946
2-5	**1.04** (1.02-1.06)	**0.98** (0.96-0.99)	**1.04** (1.02-1.05)	**0.95** (0.94-0.97)	**1.04** (1.02-1.06)	**0.91** (0.89-0.93)
6-8	**1.13** (1.11-1.15)	**1.01** (1.07-1.12)	**1.12** (1.11-1.14)	**1.06** (1.04-1.07)	**1.13** (1.11-1.15)	**1.04** (1.01-1.06)
9-11	**1.26** (1.24-1.27)	**1.22** (1.20-1.24)	**1.25** (1.23-1.27)	**1.17** (1.15-1.18)	**1.25** (1.24-1.27)	**1.14** (1.12-1.16)
12-17	**1.43** (1.41-1.45)	**1.41** (1.39-1.43)	**1.42** (1.41-1.44)	**1.31** (1.29-1.33)	**1.43** (1.41-1.45)	**1.29** (1.27-1.32)
18-19	**1.34** (1.32-1.36)	**1.33** (1.30-1.35)	**1.33** (1.31-1.36)	**1.25** (1.23-1.27)	**1.34** (1.32-1.36)	**1.24** (1.22-1.27)
20-22	**1.30** (1.28-1.33)	**1.29** (1.26-1.31)	**1.29** (1.27-1.32)	**1.20** 81.18-1.22)	**1.30** (1.28-1.33)	**1.19** (1.16-1.21)
22 and over	**1.16** (1.15-1.18)	**1.20** (1.18-1.22)	**1.16** (1.14-1.18)	**1.02** (1.00-1.03)	**1.16** (1.14-1.18)	**1.06** (1.04-1.08)
	**Wave 1**					
n of cases	10 937	8 034	10 937	10 110	10 937	7 355
2-5	0.95 (0.88-1.02)	0.92 (0.84-1.02)	0.95 (0.88-1.02)	**0.90** (0.83-0.97)	0.95 (0.88-1.02)	**0.89** (0.80-0.98)
6-8	0.96 (0.90-1.03)	0.97 (0.89-1.06)	0.96 (0.89-1.03)	**0.92** (0.85-0.98)	0.96 (0.90-1.03)	0.94 (0.85-1.02)
9-11	1.01 (0.95-1.07)	1.01 (0.94-1.08)	1.01 (0.95-1.07)	0.96 (0.90-1.02)	1.01 (0.95-1.07)	0.96 (0.89-1.04)
12-17	**1.20** (1.14-1.26)	**1.17** (1.10-1.24)	**1.19** (1.13-1.26)	**1.13** (1.07-1.19)	**1.19** (1.13-1.25)	**1.10** (1.04-1.17)
18-19	**1.34** (1.26-1.43)	**1.38** (1.28-1.48)	**1.34** (1.26-1.43)	**1.26** (1.18-1.35)	**1.34** (1.25-1.43)	**1.30** (1.21-1.40)
20-22	**1.30** (1.21-1.39)	**1.31** (1.22-1.42)	**1.29** (1.20-1.38)	**1.23** (1.15-1.32)	**1.29** (1.20-1.38)	**1.23** (1.14-1.34)
22 and over	**1.17** (1.10-1.23)	**1.22** (1.15-1.30)	**1.16** (1.10-1.23)	**1.08** (1.02-1.15)	**1.16** (1.10-1.23)	**1.13** (1.05-1.21)
	**Wave 2**					
n of cases	81 923	59 038	81 923	77 276	81 923	55 142
2-5	1.01 (0.99-1.04)	**0.96** (0.93-0.98)	1.01 (0.98-1.03)	**0.92** (0.90-0.95)	1.01 (0.99-1.04)	**0.88** (0.85-0.91)
6-8	**1.06** (1.03-1.08)	1.02 (0.99-1.05)	**1.05** (1.03-1.08)	0.99 (0.97-1.01)	**1.06** (1.03-1.08)	**0.96** (0.93-0.99)
9-11	**1.20** (1.17-1.22)	**1.15** (1.12-1.18)	**1.20** (1.17-1.22)	**1.11** (1.09-1.14)	**1.20** (1.17-1.22)	**1.08** (1.05-1.11)
12-17	**1.37** (1.35-1.40)	**1.35** (1.32-1.37)	**1.36** (1.34-1.39)	**1.25** (1.23-1.28)	**1.37** (1.34-1.39)	**1.24** (1.21-1.26)
18-19	**1.28** (1.25-1.31)	**1.26** (1.22-1.30)	**1.27** (1.24-1.30)	**1.20** (1.17-1.23)	**1.27** (1.25-1.31)	**1.18** (1.15-1.22)

20-22	**1.27** (1.24-1.31)	**1.26** (1.22-1.30)	**1.26** (1.23-1.29)	**1.17** (1.14-1.20)	**1.27** (1.24-1.30)	**1.16** (1.13-1.20)
22 and over	**1.17** (1.15-1.20)	**1.21** (1.18-1.24)	**1.17** (1.14-1.19)	**1.03** (1.01-1.05)	**1.17** (1.15-1.20)	**1.06** (1.03-1.09)
	**Wave 3**					
n of cases	96 586	68 431	96 586	91 825	96 586	64 449
2-5	**1.06** (1.04-1.08)	1.00 (0.97-1.03)	**1.06** (1.03-1.08)	0.99 (0.97-1.01)	**1.06** (1.04-1.08)	**0.94** (0.91-0.97)
6-8	**1.18** (1.16-1.21)	**1.16** (1.12-1.18)	**1.18** (1.15-1.20)	**1.12** (1.09-1.14)	**1.18** (1.16-1.21)	**1.10** (1.07-1.13)
9-11	**1.28** (1.26-1.31)	**1.25** (1.23-1.28)	**1.28** (1.26-1.31)	**1.20** (1.18-1.22)	**1.28** (1.26-1.30)	**1.18** (1.16-1.21)
12-17	**1.42** (1.40-1.45)	**1.41** (1.38-1.44)	**1.42** (1.40-1.44)	**1.32** (1.30-1.34)	**1.42** (1.40-1.45)	**1.31** (1.28-1.34)
18-19	**1.33** (1.30-1.36)	**1.31** (1.28-1.35)	**1.32** (1.29-1.35)	**1.24** (1.22-1.27)	**1.33** (1.30-1.36)	**1.24** (1.21-1.27)
20-22	**1.28** (1.25-1.31)	**1.25** (1.22-1.29)	**1.27** (1.24-1.30)	**1.18** (1.15-1.21)	**1.28** (1.25-1.31)	**1.17** (1.13-1.20)
22 and over	**1.13** (1.10-1.15)	**1.17** (1.14-1.19)	**1.12** (1.10-1.15)	0.99 (0.98-1.02)	**1.13** (1.10-1.15)	**1.04** (1.01-1.06)

For peer review only

BMJ Open

Children in the household and risk of severe COVID-19 during the first three waves of the pandemic: a prospective register study of 1.5 million Swedish men.

Journal:	*BMJ Open*
Manuscript ID	bmjopen-2022-063640.R1
Article Type:	Original research
Date Submitted by the Author:	08-Jul-2022
Complete List of Authors:	af Geijerstam, Agnes; University of Gothenburg Institute of Medicine, School of Public Health and Community Medicine<br>Mehlig, Kirsten; University of Gothenburg Institute of Medicine, School of Public Health and Community Medicine<br>Hunsberger, Monica; University of Gothenburg Institute of Medicine, School of Public Health and Community Medicine<br>Åberg, Maria; University of Gothenburg Institute of Medicine, School of Public Health and Community Medicine; Region Västra Götaland, Regionhälsan<br>Lissner, Lauren; University of Gothenburg Institute of Medicine, School of Public Health and Community Medicine,
**Primary Subject Heading**:	Epidemiology
Secondary Subject Heading:	Public health, Infectious diseases
Keywords:	COVID-19, Epidemiology < INFECTIOUS DISEASES, EPIDEMIOLOGY

SCHOLARONE™
Manuscripts

I, the Submitting Author has the right to grant and does grant on behalf of all authors of the Work (as defined in the below author licence), an exclusive licence and/or a non-exclusive licence for contributions from authors who are: i) UK Crown employees; ii) where BMJ has agreed a CC-BY licence shall apply, and/or iii) in accordance with the terms applicable for US Federal Government officers or employees acting as part of their official duties; on a worldwide, perpetual, irrevocable, royalty-free basis to BMJ Publishing Group Ltd ("BMJ") its licensees and where the relevant Journal is co-owned by BMJ to the co-owners of the Journal, to publish the Work in this journal and any other BMJ products and to exploit all rights, as set out in our licence.

The Submitting Author accepts and understands that any supply made under these terms is made by BMJ to the Submitting Author unless you are acting as an employee on behalf of your employer or a postgraduate student of an affiliated institution which is paying any applicable article publishing charge ("APC") for Open Access articles. Where the Submitting Author wishes to make the Work available on an Open Access basis (and intends to pay the relevant APC), the terms of reuse of such Open Access shall be governed by a Creative Commons licence – details of these licences and which Creative Commons licence will apply to this Work are set out in our licence referred to above.

Other than as permitted in any relevant BMJ Author's Self Archiving Policies, I confirm this Work has not been accepted for publication elsewhere, is not being considered for publication elsewhere and does not duplicate material already published. I confirm all authors consent to publication of this Work and authorise the granting of this licence.

For peer review only

Children in the household and risk of severe COVID-19 during the first three waves of the pandemic: a prospective register study of 1.5 million Swedish men.

Authors: Agnes af Geijerstam [a], Kirsten Mehlig [a], Monica Hunsberger [a], Maria Åberg [a, b], Lauren Lissner [a]

[a] School of Public Health and Community Medicine, Institute of Medicine, Sahlgrenska Academy, University of Gothenburg, Gothenburg, Sweden

[b] Region Västra Götaland, Regionhälsan, Gothenburg, Sweden

Corresponding author: Agnes af Geijerstam, School of Public Health and Community Medicine, Institute of Medicine, Sahlgrenska Academy, University of Gothenburg, P.O Box 454, 405 30 Gothenburg, Sweden. agnes.af.geijerstam@gu.se

Keywords: COVID-19, SARS-CoV-2, children, fathers, household, hospitalisation, infection, school closures, cardiorespiratory fitness, adolescence, men, population-based, cohort.

Abstract

Objective To investigate whether Swedish men living with children had elevated risk for severe COVID-19 or infection with SARS-CoV-2 during the first three waves of the pandemic.

Design Prospective registry-based cohort study.

Participants 1 557 061 Swedish men, undergoing military conscription between 1968 and 2005 at a mean age of 18.3 (SD 0.73) years.

Main outcome measures Infection with SARS-CoV-2 and hospitalisation due to COVID-19 from March 2020 to September 2021.

Results There was a protective association between pre-school children at home and hospitalisation due to COVID-19 during the first and third waves compared to only older or no children at all, with odds ratios (OR) 95% confidence limits (CI) 0.64 (0.46-0.88) and 0.73 (0.58-0.91) respectively. No association was observed for living with children 6-12 years old, but for 13-17 years old the risk increased. Age in 2020 did not explain these associations. Further adjustment for socioeconomic and health factors did not attenuate the results. Exposure to pre-school children also had a protective association with testing positive with SARS-CoV-2, with or without hospitalisation, OR = 0.91 (95% CI 0.89-0.93), while living with children of other ages was associated with increased odds of infection.

Conclusions Cohabiting with pre-school children was associated with reduced risk for severe COVID-19. Living with school-age children between 6 and 12 years had no association with severe COVID-19, but sharing the household with teenagers and young adults was associated with elevated risk. Our results

For peer review only

are of special interest since pre-schools and compulsory schools (age 6-15) in Sweden were not closed in 2020.

Strengths and limitations to this study

As schools were not closed in Sweden, the effects of living with children in this country constitutes an important comparison point.

Our large study population, based on validated registry data and including key covariates reflecting the conscript's comorbidities and physical condition, is an important strength.

The major limitation is that only men were included.

Due to the observational design, we are also unable to rule out that unknown factors influenced the results.

For peer review only

Background

Early evidence showed that children were less affected by the SARS-CoV-2 virus than adults and adolescents(1,2). This notwithstanding, with the initial attempts to curb transmission by school closures and lockdowns, children around the world saw their lives changed. The effects of school closures on the spread of infectious disease had been discussed before the COVID-19 pandemic. Modelling studies focusing on influenza showed the effectiveness varied depending on the basic reproduction number and on whether children were driving the attack rates due to less immunity compared to adults(3–5). Despite the conflicting evidence on the effectiveness of school closures in relation to the character of SARS-CoV-2, it was widely implemented as a non-pharmaceutical intervention (NPI).

Sweden was an exception where compulsory schools (ages 6-15) and pre-schools were kept open. Attendance was mandatory and enforced. Schools and pre-schools were to implement preventive measures such as distancing and hand hygiene, and avoid unnecessary mixing of classes and teachers(6), while teaching in upper secondary school was moved online.

Two large studies have been published on the risk of parental infection posed by living with children. Wood et al with a population of over 300 000 health care workers and the adults they live with in Scotland(7), and Forbes et al with the COVID-19: OpenSAFELY cohort of 12 million adults in England(8). Neither could show an increased risk related to living with children of any age during wave 1. During wave 2 there was a small absolute risk associated with children of any age living at home, except for younger children as reported in the Scottish cohort. Part of the risk increase was attributed to the return to schools and preschools in September 2020.

Men are disproportionally affected by COVID-19, comprising 74% of those admitted to intensive care in Sweden(9). In the present study, we had the opportunity to examine a large part of the male population in Sweden, for whom information is available on early health factors that influence severity of COVID-19(10,11). As schools and preschools were open in Sweden, it is a unique starting point for investigating the effects of sharing a household with children during the pandemic.

Methods

Study design

This is a prospective cohort study based on data from the Swedish Military Conscription Registry, combined with a socioeconomic population registry (LISA) from Statistics Sweden as well as the Swedish national hospital and intensive care registries.

Population studied

The Swedish military conscript registry contains information about 1 949 891 Swedish individuals who enlisted for military service between late 1968 and 2005. During those years Swedish law required all male

For peer review only

citizens to enlist, except for those in prison or those with severe chronic somatic or psychiatric conditions or functional disabilities (approximately 2-3% annually).

Patient and public involvement statement

As this is a registry-based study, there have been no patient or public involvement.

Main independent variables

Children at home

Data about children registered at the same address as the men in the cohort was retrieved from Statistics Sweden. Most of the men are assumed to be fathers, biological or not, but they could also be grandfathers or lodgers. The age brackets correspond to Swedish pre-school, primary school, middle school, and lower and upper secondary school. No children in the youngest bracket would have started school during 2020-2021 but those aged 6 in 2020 would have been in pre-school during wave 1 and then in first grade during wave 2 and 3. School in Sweden is compulsory from 6 to 15 years of age and about 85% of all 16–18-year-olds attend upper secondary school. About 90% of all Swedish 2-year-olds attend pre-school, with even higher rates for 4-5 year-olds(12).

Confounding variables

Weight and height were measured by standard anthropometric measurement techniques, and continuous BMI values (kg/m^2) were calculated, as BMI has been showed to be one of the major risk factors for severe COVID-19. Earlier studies on the same cohort have shown an association between BMI and cardiorespiratory fitness (CRF) in early adulthood and later risk of severe disease (11,13–15). There is also a known correlation between CRF, height and BMI at conscription and the probability of having children (16).

Morbidity at baseline

All medical diagnoses prior to conscription are recorded in the conscript registry. Illness that could have affected the ability or decision to have children, as well as later risk of severe COVID-19 was controlled for using ICD-codes (International Classification of Diseases) for respiratory disease, cardiovascular disease, diabetes, kidney disease and malignant cancers (16,17).

Socioeconomic indicators

Parental education was considered a proxy for socioeconomic position of the household. Using data from the LISA registry, it was based on the highest in the household and categorized as: low (up to 9 years), medium (upper secondary school diploma with ≤2 years at university) and high education (≥3 years at university). Data on home municipality was collapsed into three categories – large, medium and small towns and the municipalities nearby(18). Disposable family income was categorized into low, medium, and high income based on tertiles.

For peer review only

From LISA we also had information on profession. High risk occupation was defined a posteriori as those where the risk of hospitalisation due to COVID-19 was 0.28% and higher. This included health care personnel, bus drivers, restaurant workers, service personnel, industrial workers, social workers and primary school teachers (N=314 834).

Analytic sample

Originally comprising 1 949 891 conscripts, the cohort was reduced to 1 559 187 men after exclusions (Figure 1). 1 557 061 had information in LISA on children at home. Those who died during 2020 and until February 2021 (n=5 012) were censored in the analysis prior to each wave, giving an at risk population of 1 555 835 at the beginning of wave 1, 1 552 040 at wave 2 and 1 549 514 at wave 3. Characteristics of the population, together with crude outcome data, are shown in Table 1.

Figure 1 here

Table 1: Characteristics of the study population.

Conscription year	*1968-1975*	*1976-1985*	*1986-1995*	*1996-2005*	*All decades*
N in 2020	282 828	440 991	495 775	337 467	1 557 061
Age at conscription, mean (SD)	18.5 (0.65)	18.3 (0.82)	18.3 (0.76)	18.2 (0.58)	18.3 (0.73)
Age in 2020 mean (SD)	65.9 (1.94)	57.5 (2.98)	48.0 (3.00)	38.3 (2.91)	51.8 (9.89)
	Children at home (age in 2018)				
Child of any age	43 220 (15.3)	177 630 (40.3)	333 582 (67.3)	212 025 (62.8)	790 604 (50.8)
0-3 years (%)	292 (0.1)	3 151 (0.7)	38 873 (7.9)	111 926 (33.2)	154 242 (9.9)
4-6 years (%)	457 (0.2)	5 657 (1.3)	62 168 (12.5)	92 332 (27.4)	160 612 (10.3)
7-10 years (%)	1 275 (0.5)	16 576 (3.8)	124 800 (25.2)	79 418 (23.5)	222 069 (14.3)
11-15 years (%)	3 967 (1.4)	47 911 (10.9)	168 507 (34)	35 318 (10.5)	255 703 (16.4)
16-17 years (%)	3 597 (1.3)	35 946 (8.2)	68 234 (13.8)	5 549 (1.6)	113 326 (7.3)
18-20 years (%)	4 719 (1.7)	40 733 (9.2)	50 320 (10.2)	2 641 (0.8)	98 413 (6.3)
20 and older (%)	34 259 (12.1)	94 456 (21.4)	51 782 (10.4)	15 983 (4.7)	196 480 (12.6)
	Hospitalisations due to COVID-19				
N, March 2020-Sep 2021 (%)	2 261 (0.80)	3 179 (0.72)	2 455 (0.50)	760 (0.23)	8 655 (0.56)
With children at home (%)	426 (18.8)	1 318 (41.5)	1 616 (65.8)	463 (61)	3 823 (44.2)
	PCR confirmed infection with SARS-CoV-2				
N, March 2020-Sep 2021(%)	18 471 (6.5)	50 272 (11.4)	72 878 (14.7)	47 825 (14.2)	189 446 (12.2)
With children at home (%)	3 793 (20.5)	24 279 (48.3)	54 920 (75.4)	32 671 (68.3)	115 663 (61.1)

Outcome variables

Hospitalisation due to Covid-19

Using the Swedish personal identification number, the full sample was linked to the National Patient Register and the Swedish Intensive Care Registry. From these, all cases between March 2020 and September 2021 with a main diagnosis of ICD U071 for test verified infection with SARS-CoV-2 and U072 for clinically diagnosed COVID-19 were identified. Records with U071 or U072 as a secondary diagnosis were counted as cases if the main diagnosis was clinically related to COVID-19 (table S1). All hospital-care requiring illness was considered severe COVID-19. Register data based on Swedish hospital records have high validity (19).

Infection with COVID-19

Free PCR-testing began in summer 2020. Previously, testing was mainly done in hospitals. Therefore, data from this period is limited and not representative of the actual infection rates, as is seen in the comparison between testing and hospitalisations in Figure 2. All positive tests were to be registered in the Sminet registry according to the Swedish Communicable Diseases Act. Data from Sminet was extracted on September 21st, 2021 and covers all positives until that day.

Figure 2 here

Statistical analysis

The main independent variable was analysed as a binary variable in each age interval. Logistic regression was used to calculate the odds for hospitalisation and infection due to COVID-19 by this exposure category, adjusted for children in other age brackets, place of residence, income and profession in 2018, linear, quadratic, and cubic terms of BMI, as well as CRF, height, parental education and chronic disease at conscription. All regression models were adjusted for exact age at conscription and year of conscription examination, and thus indirectly for age in 2020. Because overall events of hospitalisation were rare, we used penalized likelihood estimation (Firth method) to reduce (potential) small-sample bias in maximum likelihood estimation

Statistical analyses were performed with SAS version 9.4 (SAS Institute, Cary, NC). Statistical significance was set at 0.05 (2-sided tests).

Ethics approval statement

The study conforms to the principles outlined in the Declaration of Helsinki. The Ethics Committee of the University of Gothenburg and Confidentiality Clearance at Statistics Sweden approved the study (EPN Reference numbers EPN 462-14 and 567-15; T174-15, T653-17, T196-17, T2019-05875, T 2020-01325, T2020-02420, T2021-00797, T2021-03310). The requirement for informed consent was waived by

For peer review only

the Ethics Committee of the University of Gothenburg for secondary analysis of existing data. The data was pseudonymized before being accessed by the study authors.

Results

Hospitalisations

Having a child of preschool age at home had a protective association with hospitalisation due to COVID-19 during the pandemic from March 2020 to July 2021 (Figure 3). This association is statistically significant in the first and third wave with OR=0.64 (95% CI 0.46-0.88) and 0.73 (0.58-0.91). During the second wave, no association could be seen (OR=1.06 (0.80-1.41)). In contrast, no associations between living with children of primary school age and hospitalisation were observed. Sharing the household with children 13 years of age or older conveyed an overall excess risk of hospitalisation.

Figure 3 here

None of the results was attenuated after adjustment for covariates (table S2). Exposures describing children of different age groups were not mutually exclusive, as many fathers live with more than one child. For the combined waves, the protective association between the youngest children and hospitalisation due to COVID-19 was strengthened to OR 0.54 (95% CI 0.42-0.71) when we considered a separate category of those with only pre-school aged children (table S3).

Infection

Living in a household with children over 5 was associated with an increased risk for infection with SARS-CoV-2. The same pattern can be seen for all waves, with odds ratios significantly higher for all age groups apart from the pre-school children who had a significant protective association (Figure 4) with OR=0.91 (95% CI 0.89-0.93). Wave specific results can be found in Supplementary table S4.

Figure 4 here

Adjustment for high-risk profession in the main analysis did not attenuate the effect estimates. To evaluate the effects of isolating with children not yet in compulsory school we examined the subset of men in high-risk occupations only: having younger children still gave a protective association with OR= 0.94 (95% CI 0.91-0.98). Again, when looking at those living with pre-school children only, the association was stronger with OR=0.73 (95% CI 0.69-0.77).

Discussion

For peer review only

In this paper we demonstrate a robust protective association between residing with children of pre-school age and the risk of severe COVID-19 during two of the three waves of the pandemic in Sweden.

The pattern for the older children varied, but no significant associations were seen at any time among those aged 6-12 years. Exposure to the age group 13-17 years was associated with a higher risk of severe COVID-19 during the second and third waves. Sharing a household with a teenager or young adult was associated with a higher risk of disease during all waves.

There were significantly higher odds for testing positive for SARS-CoV-2 associated with living with children 6 years and older, except for the age group 6-8 during the second wave. The age group 13-17 presents the largest risk for infection and includes both children who attended school (age 13-15) and who had distance learning (age 16-17). It has been shown that adolescents transmit COVID-19 disease similarly/comparably to adults in households, and the combination of slightly older children in open schools could explain this pattern (7,20–26). Unfortunately, the testing data does not include negative results, therefore it is not possible to analyse whether the infection rates partly mirror an increased testing frequency in certain groups.

The comparable studies from Scotland and England showed that having children at home (any age in England, 0-11 in Scotland) was not associated with increased risk of infection or severe disease in spring 2020. During the second wave risk of infection and COVID-19 related hospital admission was increased in the OpenSAFELY cohort but not in the Scottish cohort(7,8). This finding was attributed in part to the school closures during spring and the reopening after summer. Our results fit the same pattern but cannot be explained the same way, since the schools were open. An earlier Swedish study presents a similar small increase in infections among parents of lower-secondary school age children during the first wave(27).

High-risk profession did not affect the associations. With our smaller age brackets, we were able to single out associations in parents of children in pre-school, which were distinctly different from those with children of older ages and highlights a methodological strength of our study.

No national figures of school or pre-school attendance are available, but in the numbers of the official statistics bureau of Gothenburg (pop. 583 056) school attendance was distinctly lower in March 2020, and sick leave was higher during the pandemic compared to 2019. The possibility to self-isolate with younger children, together with more social distancing and better use of other protective measures in these families could of course contribute to the protective association, particularly considering that the effect was more pronounced in those with no older children. The Swedish pre-school teacher's union reported a large drop in attendance in March 2020. After March though, the majority returned to pre-school (28), making it unlikely that a large proportion of parents were isolating with their children. The fact that few of the pre-school fathers also lived with children older than 13 does limit their exposure to infection due to transmission in school, which also could contribute to the protective association. When the model is

For peer review only

unadjusted for older children the association is stronger with OR=0.85 (95% CI 0.83-0.87), but it is still significant in the main model where the presence or lack of older children is accounted for.

Our large study population, based on validated registry data and including key covariates reflecting the conscript's comorbidities and physical condition, is an important strength. However, since this is an observational study, we cannot rule out that unknown factors such as behavioural differences between those living with or without children of different ages influenced the results. The major limitation is that only men were included in this study. Furthermore, all information from the LISA registry is from 2018. However, only minor changes in these covariates can be expected during 2019-2020, except for the oldest children who might have left home during this period.

We were not able to include the effects of vaccination, but as the main part of our cohort were vaccinated from May 2021, the potential effect is restricted to the tapering off of wave 3, with very few cases overall.

Clinical and public health implications

The decision to keep mandatory schools open in Sweden constitute a comparison point. A recent review of studies trying to evaluate the effect of school closures on community transmission concluded that "The true independent effect of school closures from the first wave around the world may simply be unknowable"(29). The model calculations included in the review all had problems differentiating between NPI's implemented simultaneously. Closing schools was meant to control community transmission by limiting transmission between children and subsequently between children and parents. As our study shows, the odds ratio for infection was higher for men living with children of all age groups except those aged 2-5. This could be expected due to the greater number of contacts in school but is still comparable to transmission patterns where schools were closed during spring 2020.

The finding that living with children aged 2-5 was associated with lower risk of hospitalization due to COVID-19 does raise questions. If the effect is not entirely due to behavioural differences or parental health it could be speculated that simultaneous infection with other respiratory viruses more commonly occurring in this group compared to older children(30), such as rhinovirus, could be protective, as has been shown in vitro(31,32). Both wave 1 and wave 3 coincide with the months when the Swedish Social Insurance Agency normally distributes the most compensation for care of sick child (mainly due to cold viruses in early spring).

Conclusion

Young children seem to have played a minor part in the community transmission of COVID-19, even though pre-schools remained open in Sweden. As this study shows, this age group conveyed a protective association both with the risk of infection and with the risk of severe sickness in adult men living in the same household. Having children between 6 and 12 years in the household was associated with a small

For peer review only

increase in odds of infection, but not with severe disease. Having teenagers in the household was associated with increased rates of infection as well as severe disease in their fathers. These associations are similar in magnitude to those reported in other settings where schools were closed.

For peer review only

Statements

Contributorship statement: AG, LL and MÅ initiated the project. AG and KM performed all statistical analyses. AG had main responsibility for writing the article. LL, MÅ, KM and MH contributed to the structure and content of the manuscript and have read and approved of the final draft. LL and MÅ share last authorship.

Competing interest statement: All authors have completed the Unified Competing Interest form (available on request from the corresponding author) and declare: no support from any other than above mentioned organization for the submitted work; no financial relationships with any organizations that might have an interest in the submitted work in the previous three years, no other relationships or activities that could appear to have influenced the submitted work.

Funding: This work was supported by the EpiLife-Teens Research Program (FORMAS-2012-00038), by the Swedish ALF-agreement (ALFGBG-720201 and ALFGBG-813511), the Swedish Research Council (02508, VRREG 2019-00193, 2020-05792) and Swedish Research Council for Health, Working Life and Welfare (2021-00304). The funding sources had no role in study design, collection, analysis and interpretation of data, the writing of the report, or the decision to submit the article for publication.

Data sharing statement: The data used in this study is available upon request from The Swedish Defence Conscription and Assessment Agency and the National Board of Health and Welfare.

Transparency statement: AG affirms that the manuscript is an honest, accurate, and transparent account of the study being reported and that no important aspects of the study have been omitted.

Dissemination to participants and related patient and public communities: We will make the findings of this article public with a press release and via media contacts.

For peer review only

References

1. Viner RM, Mytton OT, Bonell C, Melendez-Torres G, Ward J, Hudson L, et al. Susceptibility to SARS-CoV-2 infection among children and adolescents compared with adults: a systematic review and meta-analysis. JAMA pediatrics. 2021;175(2):143–56.

2. Ludvigsson JF. Systematic review of COVID-19 in children shows milder cases and a better prognosis than adults. Acta paediatrica. 2020;109(6):1088–95.

3. Cauchemez S, Ferguson NM, Wachtel C, Tegnell A, Saour G, Duncan B, et al. Closure of schools during an influenza pandemic. The Lancet infectious diseases. 2009;9(8):473–81.

4. Mangtani P. Impact of school closures on an influenza pandemic: scientific evidence base review. 2014;

5. Jackson C, Mangtani P, Hawker J, Olowokure B, Vynnycky E. The effects of school closures on influenza outbreaks and pandemics: systematic review of simulation studies. PloS one. 2014;9(5):e97297.

6. Utbildningsdepartementet. Förordning (2020:115) om utbildning på skolområdet och annan pedagogisk verksamhet vid spridning av viss smitta [Internet]. Mar 13, 2020. Available from: https://www.riksdagen.se/sv/dokument-lagar/dokument/svensk-forfattningssamling/forordning-2020115-om-utbildning-i-vissa_sfs-2020-115

7. Wood R, Thomson E, Galbraith R, Gribben C, Caldwell D, Bishop J, et al. Sharing a household with children and risk of COVID-19: a study of over 300 000 adults living in healthcare worker households in Scotland. Archives of disease in childhood. 2021;

8. Forbes H, Morton CE, Bacon S, McDonald HI, Minassian C, Brown JP, et al. Association between living with children and outcomes from covid-19: OpenSAFELY cohort study of 12 million adults in England. bmj. 2021;372.

9. Public Health Agency of Sweden. Bekräftade fall i Sverige – daglig uppdatering [Internet]. Public Health Agency of Sweden; Available from: https://www.folkhalsomyndigheten.se/smittskydd-beredskap/utbrott/aktuella-utbrott/covid-19/statistik-och-analyser/bekraftade-fall-i-sverige/

10. Af Geijerstam A, Mehlig K, Börjesson M, Robertson J, Nyberg J, Adiels M, et al. Fitness, strength and severity of COVID-19: a prospective register study of 1 559 187 Swedish conscripts. BMJ open. 2021;11(7):e051316.

11. Robertson J, Adiels M, Lissner L, Mehlig K, Af Geijerstam A, Lindgren M, et al. BMI in early adulthood is associated with severe COVID-19 later in life–a prospective cohort study of 1.5 million Swedish men. Obesity. 2022;

12. Förskola – Barn och grupper – Riksnivå [Internet]. Available from: https://www.skolverket.se/skolutveckling/statistik

13. Gao M, Piernas C, Astbury NM, Hippisley-Cox J, O'Rahilly S, Aveyard P, et al. Associations between body-mass index and COVID-19 severity in 6· 9 million people in England: a prospective, community-based, cohort study. The Lancet Diabetes & Endocrinology. 2021;

For peer review only

14. Hamer M, Gale CR, Kivimäki M, Batty GD. Overweight, obesity, and risk of hospitalization for COVID-19: A community-based cohort study of adults in the United Kingdom. Proceedings of the National Academy of Sciences. 2020;117(35):21011–3.

15. Clark A, Jit M, Warren-Gash C, Guthrie B, Wang HH, Mercer SW, et al. Global, regional, and national estimates of the population at increased risk of severe COVID-19 due to underlying health conditions in 2020: a modelling study. The Lancet Global Health. 2020;8(8):e1003–17.

16. Barclay K, Kolk M. The influence of health in early adulthood on male fertility. Population and Development Review. 2020;46(4):757–85.

17. Glazer CH, Bonde JP, Eisenberg ML, Giwercman A, Hærvig KK, Rimborg S, et al. Male infertility and risk of nonmalignant chronic diseases: a systematic review of the epidemiological evidence. In Thieme Medical Publishers; 2017. p. 282–90.

18. Sveriges kommuner och regioner. Kommungruppsindelning [Internet]. Available from: https://skr.se/skr/tjanster/kommunerochregioner/faktakommunerochregioner/kommungruppsindelning.2051.html

19. Ludvigsson JF, Andersson E, Ekbom A, Feychting M, Kim JL, Reuterwall C, et al. External review and validation of the Swedish national inpatient register. BMC public health. 2011;11(1):1–16.

20. Madewell ZJ, Yang Y, Longini IM, Halloran ME, Dean NE. Household Transmission of SARS-CoV-2: A Systematic Review and Meta-analysis. JAMA network open. 2020;3(12):e2031756–e2031756.

21. Soriano-Arandes A, Gatell A, Serrano P, Biosca M, Campillo F, Capdevila R, et al. Household SARS-CoV-2 transmission and children: a network prospective study. Clinical Infectious Diseases: an Official Publication of the Infectious Diseases Society of America. 2021;

22. Li F, Li YY, Liu MJ, Fang LQ, Dean NE, Wong GW, et al. Household transmission of SARS-CoV-2 and risk factors for susceptibility and infectivity in Wuhan: a retrospective observational study. The Lancet Infectious Diseases. 2021;21(5):617–28.

23. Brandal LT, Ofitserova TS, Meijerink H, Rykkvin R, Lund HM, Hungnes O, et al. Minimal transmission of SARS-CoV-2 from paediatric COVID-19 cases in primary schools, Norway, August to November 2020. Eurosurveillance. 2021;26(1):2002011.

24. Goldstein E, Lipsitch M, Cevik M. On the Effect of Age on the Transmission of SARS-CoV-2 in Households, Schools, and the Community. The Journal of infectious diseases. 2021;223(3):362–9.

25. Telle K, Jørgensen SB, Hart R, Greve-Isdahl M, Kacelnik O. Secondary attack rates of COVID-19 in Norwegian families: A nation-wide register-based study. European journal of epidemiology. 2021;1–8.

26. Dattner I, Goldberg Y, Katriel G, Yaari R, Gal N, Miron Y, et al. The role of children in the spread of COVID-19: Using household data from Bnei Brak, Israel, to estimate the relative susceptibility and infectivity of children. PLoS computational biology. 2021;17(2):e1008559.

27. Vlachos J, Hertegård E, Svaleryd HB. The effects of school closures on SARS-CoV-2 among parents and teachers. Proceedings of the National Academy of Sciences. 2021;118(9).

For peer review only

28. Fock C. Coronatapp: 9 av 10 förskolebarn hölls hemma. Förskolan [Internet]. 2020 Jul 6; Available from: https://www.lararen.se/forskolan/coronaviruset/coronatapp-9-av-10-forskolebarn-holls-hemma

29. Walsh S, Chowdhury A, Braithwaite V, Russell S, Birch JM, Ward JL, et al. Do school closures and school reopenings affect community transmission of COVID-19? A systematic review of observational studies. BMJ Open. 2021 Aug 1;11(8):e053371.

30. Monto AS. Epidemiology of viral respiratory infections. The American journal of medicine. 2002;112(6):4–12.

31. Dee K, Goldfarb DM, Haney J, Amat JA, Herder V, Stewart M, et al. Human rhinovirus infection blocks SARS-CoV-2 replication within the respiratory epithelium: implications for COVID-19 epidemiology. Journal of Infectious Diseases. 2021;

32. Kuitunen I, Artama M, Haapanen M, Renko M. Rhinovirus spread in children during the COVID-19 pandemic despite social restrictions—A nationwide register study in Finland. Journal of Medical Virology. 2021;93(10):6063–7.

For peer review only

Legends

Figure 1: Creation of analytical sample.

Table 1: Characteristics of the study population.

Figure 2: Weekly admissions to hospital due to COVID-19 in Sweden and weekly registered PCR-verified infections between March 2020 and September 2020. Note that tests were not widely available until July 2020.

Figure 3: Associations between children in the household and hospitalisation due to COVID-19 (n=1 557 061). Odds ratios with 95% CI. Model controlled for children in other age groups, age, baseline BMI, CRF, height, chronic morbidity, parental education, income, profession and place of residence in 2018.

Figure 4: Associations between children in the household and testing positive for SARS-CoV-2 March 2020-September 2021 (n=1 557 061). Odds ratios with 95% CI. Model controlled for conscript's age, BMI, CRF, height, chronic morbidity, parental education and place of residence in 2018.

For peer review only

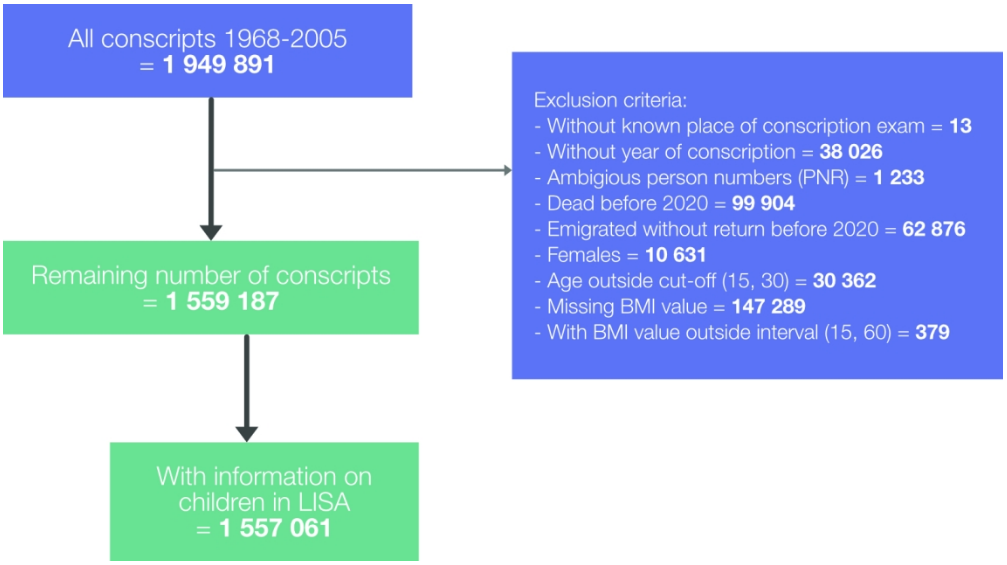

136x76mm (300 x 300 DPI)

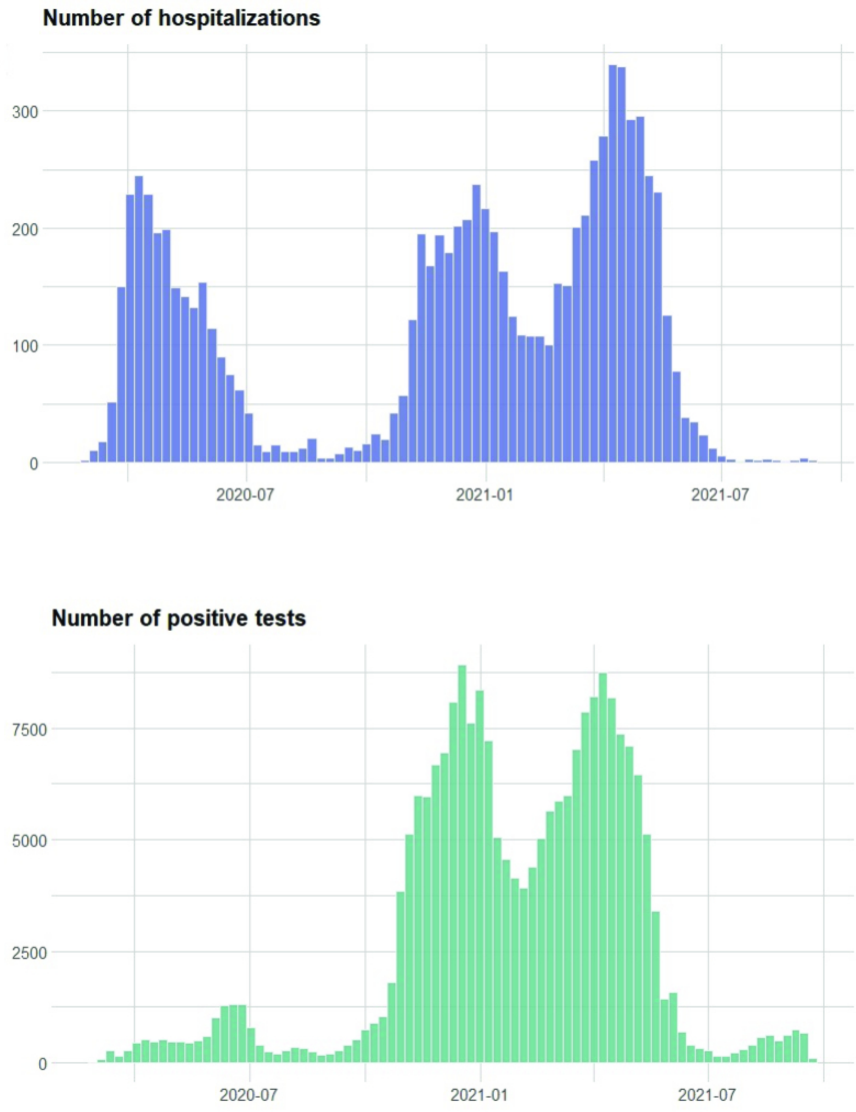

72x92mm (300 x 300 DPI)

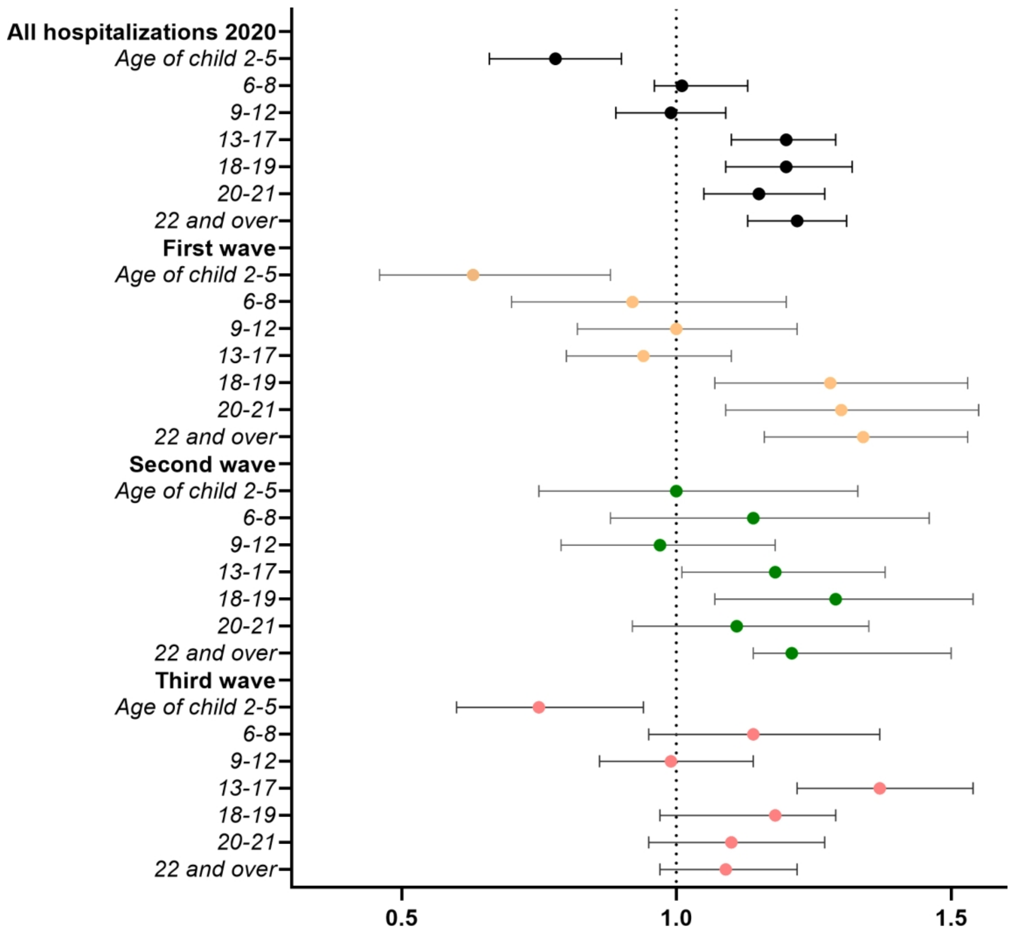

166x152mm (300 x 300 DPI)

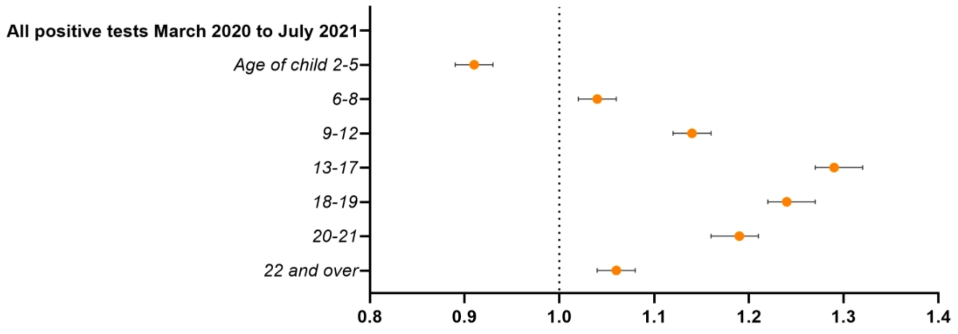

215x79mm (300 x 300 DPI)

Table S1. Included main diagnoses divided by categories when COVID-19 is secondary diagnosis.

Included categories	ICD-codes
**COVID-related symptoms**	
Cough	R05
Abnormalities of breathing	R06
Pain in throat and chest	R07
Other symptoms and signs involving the circulatory and respiratory system	R09
Dizziness and giddiness	R42
Fever of other or unknown origin	R50
Headache	R51
Malaise and fatigue	R53
Syncope and collapse	R55
**Upper and lower respiratory tract infections**	
Acute nasopharyngitis	J00
Acute tonsillitis	J03
Acute upper respiratory infections of multiple and unspecified sites	J06
Influenza due to other identified influenza virus	J10
Other viral pneumonia	J128
Viral pneumonia, unspecified	J129
Pneumonia due to Streptococcus pneumoniae	J13
Bacterial pneumonia, not elsewhere classified	J15
Pneumonia due to other specified infectious organisms	J168
Pneumonia in diseases classified elsewhere	J17
Pneumonia, unspecified organism	J18
Unspecified acute lower respiratory infection	J22
Coronavirus infection, unspecified	B342
Other viral infections of unspecified site	B348
Viral infection, unspecified	B349
Coronavirus as the cause of diseases classified elsewhere	B972
Other and unspecified infectious diseases	B99
**Respiratory disorders**	
Pulmonary embolism	I26
Acute respiratory distress syndrome	J80
Pulmonary edema	J81
Pleural effusion not elsewhere classified	J90
Respiratory failure, not elsewhere classified	J96
Respiratory disorders in diseases classified elsewhere	J99
**Obstructive Airway Diseases**	
Acute bronchitis due to other specified organisms	J208
Acute bronchitis, unspecified	J209
Acute bronchiolitis due to other specified organisms	J218
Acute bronchiolitis, unspecified	J219
Other chronic obstructive pulmonary disease	J44
Asthma	J45
**Cardiac diseases**	
Viral carditis	B332
Chronic ischemic heart disease	I25
Acute pericarditis	I30
Pericarditis in diseases classified elsewhere	I32
Acute myocarditis, unspecified	I40
Myocarditis in diseases classified elsewhere	I41
Atrial fibrillation and flutter	I48
Heart failure	I50
Abnormalities of heart beat	R00

For peer review only

**Kidney disorders**	
Acute kidney failure	N17
Chronic kidney disease	N18
Unspecified kidney failure	N19
**Electrolyte disorders**	
Other disorders of fluid, electrolyte and acid-base balance	E87

For peer review only

Table S2: Result of hospitalization analysis. OR, 95% CI. Basic model includes adjusting for age in 2020 and children of other ages. Reference for each age bracket is children in other brackets or no children at all.

Age of child in 2020	*Basic model*	*Adjusted for baseline CRF*	*Adjusted for baseline BMI, height, morbidity*	*Adjusted for parental education, income, region*	*Adjusted for profession*	*Full model*
	All hospitalizations March 2020 – September 2021					
N	1 557 061	1 142 222	1 557 061	1 420 206	1 557 061	1 027 123
n of cases	8 655	6 731	8 655	7 497	8 655	5 765
2-5	**0.74** (0.66-0.83)	**0.72** (0.62-0.84)	**0.76** (0.68-0.85)	**0.78** (0.70-0.88)	**0.75** (0.66-0.84)	**0.78** (0.66-0.90)
6-8	0.95 (0.86-1.05)	1.02 (0.89-1.16)	0.96 (0.87-1.06)	0.99 (0.90-1.10)	0.95 (0.86-1.05)	1.01 (0.95-1.13)
9-12	0.98 (0.90-1.06)	0.94 (0.84-1.02)	0.99 (0.92-1.08)	1.03 (0.95-1.11)	0.98 (0.91-1.06)	0.99 (0.89-1.10)
13-17	**1.16** (1.09-1.24)	**1.16** (1.07-1.25)	**1.18** (1.10-1.26)	**1.21** (1.13-1.29)	**1.16** (1.08-1.23)	**1.19** (1.10-1.29)
18-19	**1.16** (1.07-1.25)	**1.16** (1.06-1.27)	**1.17** (1.08-1.27)	**1.18** (1.09-1.29)	**1.16** (1.07-1.26)	**1.20** (1.09-1.32)
20-22	1.09 (0.99-1.18)	1.10 (0.99-1.20)	**1.10** (1.01-1.19)	**1.14** (1.05-1.25)	1.08 (0.99-1.17)	**1.15** (1.04-1.27)
22 and over	**1.15** (1.08-1.21)	**1.17** (1.09-1.24)	**1.15** (1.08-1.21)	**1.20** (1.12-1.28)	**1.14** (1.08-1.21)	**1.20** (1.13-1.30)
	Wave 1 (March-September 2020)					
n of cases	2 430	1 884	2 430	2 096	2 430	1 612
2-5	**0.71** (0.57-0.89)	**0.60** (0.44-0.82)	**0.73** (0.58-0.91)	**0.75** (0.60-0.94)	**0.72** (0.57-0.89)	**0.64** (0.46-0.88)
6-8	0.86 (0.70-1.05)	0.86 (0.66-1.12)	0.87 (0.72-1.06)	0.91 (0.74-1.12)	0.87 (0.71-1.06)	0.91 (0.70-1.19)
9-11	0.91 (0.78-1.07)	0.91 (0.75-1.10)	0.93 (0.79-1.08)	0.99 (0.84-1.16)	0.91 (0.78-1.07)	1.00 (0.82-1.22)
12-17	0.94 (0.83-1.07)	0.90 (0.78-1.05)	0.96 (0.84-1.09)	1.00 (0.87-1.14)	0.94 (0.83-1.07)	0.94 (0.80-1.10)
18-19	1.16 (0.99-1.35)	**1.23** (1.04-1.46)	1.17 (1.00-1.37)	**1.18** (1.00-1.41)	1.15 (0.99-1.35)	**1.26** (1.05-1.51)
20-22	1.16 (0.99-1.35)	**1.22** (1.03-1.45)	1.17 (1.00-1.37)	**1.24** (1.05-1.46)	1.15 (0.99-1.34)	**1.30** (1.09-1.55)
22 and over	**1.24** (1.11-1.38)	**1.24** (1.10-1.40)	**1.24** (1.12-1.38)	**1.33** (1.18-1.51)	**1.24** (1.11-1.38)	**1.32** (1.15-1.51)
	Wave 2 (September 2020-February 2021)					
n of cases	2 575	2 041	2 575	2 182	2 575	1 707
2-5	**0.80** (0.64-0.99)	0.94 (0.72-1.24)	0.82 (0.66-1.03)	0.86 (0.68-1.08)	0.80 (0.64-1.00)	1.06 (0.80-1.41)
6-8	0.93 (0.76-1.12)	1.04 (0.81-1.33)	0.94 (0.77-1.15)	0.99 (0.80-1.21)	0.93 (0.76-1.13)	1.15 (0.90-1.48)
9-11	0.99 (0.85-1.16)	0.90 (0.75-1.11)	1.00 (0.86-1.17)	1.04 (0.88-1.22)	0.99 (0.85-1.16)	0.96 (0.78-1.17)
12-17	1.11 (0.97-1.26)	1.15 (0.99-1.32)	1.14 (1.00-1.29)	**1.17** (1.02-1.33)	1.12 (0.99-1.26)	**1.22** (1.04-1.41)
18-19	**1.20** (1.03-1.40)	1.16 (0.98-1.38)	**1.21** (1.04-1.41)	**1.28** (1.09-1.50)	**1.20** (1.03-1.40)	**1.28** (1.07-1.52)
20-22	1.01 (0.86-1.18)	1.02 (0.85-1.21)	1.02 (0.87-1.20)	1.08 (0.91-1.28)	1.01 (0.86-1.18)	1.11 (0.92-1.35)

22 and over	**1.17** (1.05-1.31)	**1.19** (1.07-1.35)	**1.17** (1.05-1.30)	**1.27** (1.12-1.42)	**1.16** (1.05-1.29)	**1.31** (1.15-1.50)
	Wave 3 (February 2021-July 2021)					
n of cases	3 650	2 806	3 650	3 219	3 650	2 446
2-5	**0.73** (0.62-0.87)	**0.69** (0.55-0.86)	**0.75** (0.64-0.89)	**0.76** (0.64-0.90)	**0.73** (0.62-0.87)	**0.73** (0.58-0.91)
6-8	1.01 (0.88-1.17)	1.10 (0.92-1.31)	1.03 (0.90-1.19)	1.05 (0.90-1.21)	1.01 (0.88-1.17)	1.14 (0.95-1.37)
9-11	1.02 (0.91-1.14)	0.97 (0.84-1.11)	1.04 (0.93-1.16)	1.04 (0.92-1.17)	1.02 (0.91-1.14)	0.99 (0.86-1.14)
12-17	**1.33** (1.21-1.46)	**1.34** (1.20-1.50)	**1.35** (1.23-1.48)	**1.36** (1.23-1.50)	**1.33** (1.21-1.46)	**1.35** (1.21-1.52)
18-19	**1.14** (1.01-1.28)	1.11 (0.97-1.27)	**1.15** (1.02-1.30)	1.13 (1.00-1.29)	**1.14** (1.01-1.29)	1.12 (0.97-1.30)
20-22	1.08 (0.96-1.23)	1.06 (0.92-1.20)	1.10 (0.97-1.24)	1.13 (0.99-1.29)	1.08 (0.96-1.23)	1.08 (0.93-1.26)
22 and over	1.07 (0.97-1.17)	1.09 (0.98-1.21)	1.07 (0.97-1.17)	1.07 (0.96-1.18)	1.07 (0.99-1.16)	1.07 (0.95-1.20)

Table S3: Associations between children in the household and hospitalization due to COVID-19. Model controlled for age, baseline BMI, CRF, height, chronic morbidity, parental education, income, profession and place of residence in 2018. OR, 95% CI.

	Only children 2-5	***Only children 6-12***	***Only children 13-22***	***Only Children 6-22***
N	66 385	119 397	382 277	698 938
n of cases	120	426	2 504	3 690
March 2020-July 2021	**0.54** (0.42-0.71)	0.95 (0.84-1.08)	**1.28** (1.21-1.36)	**1.30** (1.22-1.39)
Wave 1	**0.49** (0.29-0.82)	**0.74** (0.57-0.98)	**1.30** (1.16-1.46)	**1.22** (1.08-1.38)
Wave 2	0.60 (0.36-1.00)	0.78 (0.59-1.02)	**1.34** (1.20-1.50)	**1.37** (1.21-1.54)
Wave 3	**0.57** (0.40-0.82)	1.18 (0.99-1.40)	**1.24** (1.13-1.36)	**1.31** (1.19-1.44)

For peer review only

Table S4: Result of infection analysis. OR, 95% CI. Basic model includes adjusting for age in 2020 and children of other ages. Reference for each age bracket is children in other brackets or no children at all.

Age of child in 2020	*Basic model*	*Adjusted for baseline CRF*	*Adjusted for baseline BMI, height, morbidity*	*Adjusted for parental education, income*	*Adjusted for profession*	*Full model*
			**All test positives**			
N	1 557 061	1 142 222	1 557 061	1 420 206	1 557 061	1 027 123
n of cases	189 446	135 503	189 446	179 211	189 446	126 946
2-5	**1.04** (1.02-1.06)	**0.98** (0.96-0.99)	**1.04** (1.02-1.05)	**0.95** (0.94-0.97)	**1.04** (1.02-1.06)	**0.91** (0.89-0.93)
6-8	**1.13** (1.11-1.15)	**1.01** (1.07-1.12)	**1.12** (1.11-1.14)	**1.06** (1.04-1.07)	**1.13** (1.11-1.15)	**1.04** (1.01-1.06)
9-11	**1.26** (1.24-1.27)	**1.22** (1.20-1.24)	**1.25** (1.23-1.27)	**1.17** (1.15-1.18)	**1.25** (1.24-1.27)	**1.14** (1.12-1.16)
12-17	**1.43** (1.41-1.45)	**1.41** (1.39-1.43)	**1.42** (1.41-1.44)	**1.31** (1.29-1.33)	**1.43** (1.41-1.45)	**1.29** (1.27-1.32)
18-19	**1.34** (1.32-1.36)	**1.33** (1.30-1.35)	**1.33** (1.31-1.36)	**1.25** (1.23-1.27)	**1.34** (1.32-1.36)	**1.24** (1.22-1.27)
20-22	**1.30** (1.28-1.33)	**1.29** (1.26-1.31)	**1.29** (1.27-1.32)	**1.20** 81.18-1.22)	**1.30** (1.28-1.33)	**1.19** (1.16-1.21)
22 and over	**1.16** (1.15-1.18)	**1.20** (1.18-1.22)	**1.16** (1.14-1.18)	**1.02** (1.00-1.03)	**1.16** (1.14-1.18)	**1.06 (**1.04-1.08)
			**Wave 1**			
n of cases	10 937	8 034	10 937	10 110	10 937	7 355
2-5	0.95 (0.88-1.02)	0.92 (0.84-1.02)	0.95 (0.88-1.02)	**0.90** (0.83-0.97)	0.95 (0.88-1.02)	**0.89** (0.80-0.98)
6-8	0.96 (0.90-1.03)	0.97 (0.89-1.06)	0.96 (0.89-1.03)	**0.92** (0.85-0.98)	0.96 (0.90-1.03)	0.94 (0.85-1.02)
9-11	1.01 (0.95-1.07)	1.01 (0.94-1.08)	1.01 (0.95-1.07)	0.96 (0.90-1.02)	1.01 (0.95-1.07)	0.96 (0.89-1.04)
12-17	**1.20** (1.14-1.26)	**1.17** (1.10-1.24)	**1.19** (1.13-1.26)	**1.13** (1.07-1.19)	**1.19** (1.13-1.25)	**1.10** (1.04-1.17)
18-19	**1.34** (1.26-1.43)	**1.38** (1.28-1.48)	**1.34** (1.26-1.43)	**1.26** (1.18-1.35)	**1.34** (1.25-1.43)	**1.30** (1.21-1.40)
20-22	**1.30** (1.21-1.39)	**1.31** (1.22-1.42)	**1.29** (1.20-1.38)	**1.23** (1.15-1.32)	**1.29** (1.20-1.38)	**1.23** (1.14-1.34)
22 and over	**1.17** (1.10-1.23)	**1.22** (1.15-1.30)	**1.16** (1.10-1.23)	**1.08** (1.02-1.15)	**1.16** (1.10-1.23)	**1.13** (1.05-1.21)
			**Wave 2**			
n of cases	81 923	59 038	81 923	77 276	81 923	55 142
2-5	1.01 (0.99-1.04)	**0.96** (0.93-0.98)	1.01 (0.98-1.03)	**0.92** (0.90-0.95)	1.01 (0.99-1.04)	**0.88** (0.85-0.91)
6-8	**1.06** (1.03-1.08)	1.02 (0.99-1.05)	**1.05** (1.03-1.08)	0.99 (0.97-1.01)	**1.06** (1.03-1.08)	**0.96** (0.93-0.99)
9-11	**1.20** (1.17-1.22)	**1.15** (1.12-1.18)	**1.20** (1.17-1.22)	**1.11** (1.09-1.14)	**1.20** (1.17-1.22)	**1.08** (1.05-1.11)
12-17	**1.37** (1.35-1.40)	**1.35** (1.32-1.37)	**1.36** (1.34-1.39)	**1.25** (1.23-1.28)	**1.37** (1.34-1.39)	**1.24** (1.21-1.26)
18-19	**1.28** (1.25-1.31)	**1.26** (1.22-1.30)	**1.27** (1.24-1.30)	**1.20** (1.17-1.23)	**1.27** (1.25-1.31)	**1.18** (1.15-1.22)

20-22	**1.27** (1.24-1.31)	**1.26** (1.22-1.30)	**1.26** (1.23-1.29)	**1.17** (1.14-1.20)	**1.27** (1.24-1.30)	**1.16** (1.13-1.20)
22 and over	**1.17** (1.15-1.20)	**1.21** (1.18-1.24)	**1.17** (1.14-1.19)	**1.03** (1.01-1.05)	**1.17** (1.15-1.20)	**1.06** (1.03-1.09)
			**Wave 3**			
n of cases	96 586	68 431	96 586	91 825	96 586	64 449
2-5	**1.06** (1.04-1.08)	1.00 (0.97-1.03)	**1.06** (1.03-1.08)	0.99 (0.97-1.01)	**1.06** (1.04-1.08)	**0.94** (0.91-0.97)
6-8	**1.18** (1.16-1.21)	**1.16** (1.12-1.18)	**1.18** (1.15-1.20)	**1.12** (1.09-1.14)	**1.18** (1.16-1.21)	**1.10** (1.07-1.13)
9-11	**1.28** (1.26-1.31)	**1.25** (1.23-1.28)	**1.28** (1.26-1.31)	**1.20** (1.18-1.22)	**1.28** (1.26-1.30)	**1.18** (1.16-1.21)
12-17	**1.42** (1.40-1.45)	**1.41** (1.38-1.44)	**1.42** (1.40-1.44)	**1.32** (1.30-1.34)	**1.42** (1.40-1.45)	**1.31** (1.28-1.34)
18-19	**1.33** (1.30-1.36)	**1.31** (1.28-1.35)	**1.32** (1.29-1.35)	**1.24** (1.22-1.27)	**1.33** (1.30-1.36)	**1.24** (1.21-1.27)
20-22	**1.28** (1.25-1.31)	**1.25** (1.22-1.29)	**1.27** (1.24-1.30)	**1.18** (1.15-1.21)	**1.28** (1.25-1.31)	**1.17** (1.13-1.20)
22 and over	**1.13** (1.10-1.15)	**1.17** (1.14-1.19)	**1.12** (1.10-1.15)	0.99 (0.98-1.02)	**1.13** (1.10-1.15)	**1.04** (1.01-1.06)

For peer review only

BMJ Open

Children in the household and risk of severe COVID-19 during the first three waves of the pandemic: a prospective registry-based cohort study of 1.5 million Swedish men.

Journal:	*BMJ Open*
Manuscript ID	bmjopen-2022-063640.R2
Article Type:	Original research
Date Submitted by the Author:	11-Jul-2022
Complete List of Authors:	af Geijerstam, Agnes; University of Gothenburg Institute of Medicine, School of Public Health and Community Medicine<br>Mehlig, Kirsten; University of Gothenburg Institute of Medicine, School of Public Health and Community Medicine<br>Hunsberger, Monica; University of Gothenburg Institute of Medicine, School of Public Health and Community Medicine<br>Åberg, Maria; University of Gothenburg Institute of Medicine, School of Public Health and Community Medicine; Region Västra Götaland, Regionhälsan<br>Lissner, Lauren; University of Gothenburg Institute of Medicine, School of Public Health and Community Medicine,
**Primary Subject Heading**:	Epidemiology
Secondary Subject Heading:	Public health, Infectious diseases
Keywords:	COVID-19, Epidemiology < INFECTIOUS DISEASES, EPIDEMIOLOGY

SCHOLARONE™
Manuscripts

I, the Submitting Author has the right to grant and does grant on behalf of all authors of the Work (as defined in the below author licence), an exclusive licence and/or a non-exclusive licence for contributions from authors who are: i) UK Crown employees; ii) where BMJ has agreed a CC-BY licence shall apply, and/or iii) in accordance with the terms applicable for US Federal Government officers or employees acting as part of their official duties; on a worldwide, perpetual, irrevocable, royalty-free basis to BMJ Publishing Group Ltd ("BMJ") its licensees and where the relevant Journal is co-owned by BMJ to the co-owners of the Journal, to publish the Work in this journal and any other BMJ products and to exploit all rights, as set out in our licence.

The Submitting Author accepts and understands that any supply made under these terms is made by BMJ to the Submitting Author unless you are acting as an employee on behalf of your employer or a postgraduate student of an affiliated institution which is paying any applicable article publishing charge ("APC") for Open Access articles. Where the Submitting Author wishes to make the Work available on an Open Access basis (and intends to pay the relevant APC), the terms of reuse of such Open Access shall be governed by a Creative Commons licence – details of these licences and which Creative Commons licence will apply to this Work are set out in our licence referred to above.

Other than as permitted in any relevant BMJ Author's Self Archiving Policies, I confirm this Work has not been accepted for publication elsewhere, is not being considered for publication elsewhere and does not duplicate material already published. I confirm all authors consent to publication of this Work and authorise the granting of this licence.

For peer review only

Children in the household and risk of severe COVID-19 during the first three waves of the pandemic: a prospective registry-based cohort study of 1.5 million Swedish men.

Authors: Agnes af Geijerstam [a], Kirsten Mehlig [a], Monica Hunsberger [a], Maria Åberg [a, b], Lauren Lissner [a]

[a] School of Public Health and Community Medicine, Institute of Medicine, Sahlgrenska Academy, University of Gothenburg, Gothenburg, Sweden

[b] Region Västra Götaland, Regionhälsan, Gothenburg, Sweden

Corresponding author: Agnes af Geijerstam, School of Public Health and Community Medicine, Institute of Medicine, Sahlgrenska Academy, University of Gothenburg, P.O Box 454, 405 30 Gothenburg, Sweden. agnes.af.geijerstam@gu.se

Keywords: COVID-19, SARS-CoV-2, children, fathers, household, hospitalisation, infection, school closures, cardiorespiratory fitness, adolescence, men, population-based, cohort.

Abstract

Objective To investigate whether Swedish men living with children had elevated risk for severe COVID-19 or infection with SARS-CoV-2 during the first three waves of the pandemic.

Design Prospective registry-based cohort study.

Participants 1 557 061 Swedish men, undergoing military conscription between 1968 and 2005 at a mean age of 18.3 (SD 0.73) years.

Main outcome measures Infection with SARS-CoV-2 and hospitalisation due to COVID-19 from March 2020 to September 2021.

Results There was a protective association between pre-school children at home and hospitalisation due to COVID-19 during the first and third waves compared to only older or no children at all, with odds ratios (OR) 95% confidence limits (CI) 0.64 (0.46-0.88) and 0.73 (0.58-0.91) respectively. No association was observed for living with children 6-12 years old, but for 13-17 years old the risk increased. Age in 2020 did not explain these associations. Further adjustment for socioeconomic and health factors did not attenuate the results. Exposure to pre-school children also had a protective association with testing positive with SARS-CoV-2, with or without hospitalisation, OR = 0.91 (95% CI 0.89-0.93), while living with children of other ages was associated with increased odds of infection.

Conclusions Cohabiting with pre-school children was associated with reduced risk for severe COVID-19. Living with school-age children between 6 and 12 years had no association with severe COVID-19, but sharing the household with teenagers and young adults was associated with elevated risk. Our results

For peer review only

are of special interest since pre-schools and compulsory schools (age 6-15) in Sweden were not closed in 2020.

Strengths and limitations to this study

As schools were not closed in Sweden, the effects of living with children in this country constitutes an important comparison point.

Our large study population, based on validated registry data and including key covariates reflecting the conscript's comorbidities and physical condition, is an important strength.

The major limitation is that only men were included.

Due to the observational design, we are also unable to rule out that unknown factors influenced the results.

For peer review only

Background

Early evidence showed that children were less affected by the SARS-CoV-2 virus than adults and adolescents(1,2). This notwithstanding, with the initial attempts to curb transmission by school closures and lockdowns, children around the world saw their lives changed. The effects of school closures on the spread of infectious disease had been discussed before the COVID-19 pandemic. Modelling studies focusing on influenza showed the effectiveness varied depending on the basic reproduction number and on whether children were driving the attack rates due to less immunity compared to adults(3–5). Despite the conflicting evidence on the effectiveness of school closures in relation to the character of SARS-CoV-2, it was widely implemented as a non-pharmaceutical intervention (NPI).

Sweden was an exception where compulsory schools (ages 6-15) and pre-schools were kept open. Attendance was mandatory and enforced. Schools and pre-schools were to implement preventive measures such as distancing and hand hygiene, and avoid unnecessary mixing of classes and teachers(6), while teaching in upper secondary school was moved online.

Two large studies have been published on the risk of parental infection posed by living with children. Wood et al with a population of over 300 000 health care workers and the adults they live with in Scotland(7), and Forbes et al with the COVID-19: OpenSAFELY cohort of 12 million adults in England(8). Neither could show an increased risk related to living with children of any age during wave 1. During wave 2 there was a small absolute risk associated with children of any age living at home, except for younger children as reported in the Scottish cohort. Part of the risk increase was attributed to the return to schools and preschools in September 2020.

Men are disproportionally affected by COVID-19, comprising 74% of those admitted to intensive care in Sweden(9). In the present study, we had the opportunity to examine a large part of the male population in Sweden, for whom information is available on early health factors that influence severity of COVID-19(10,11). As schools and preschools were open in Sweden, it is a unique starting point for investigating the effects of sharing a household with children during the pandemic.

Methods

Study design

This is a prospective cohort study based on data from the Swedish Military Conscription Registry, combined with a socioeconomic population registry (LISA) from Statistics Sweden as well as the Swedish national hospital and intensive care registries.

Population studied

The Swedish military conscript registry contains information about 1 949 891 Swedish individuals who enlisted for military service between late 1968 and 2005. During those years Swedish law required all male

For peer review only

citizens to enlist, except for those in prison or those with severe chronic somatic or psychiatric conditions or functional disabilities (approximately 2-3% annually).

Patient and public involvement statement

As this is a registry-based study, there have been no patient or public involvement.

Main independent variables

Children at home

Data about children registered at the same address as the men in the cohort was retrieved from Statistics Sweden. Most of the men are assumed to be fathers, biological or not, but they could also be grandfathers or lodgers. The age brackets correspond to Swedish pre-school, primary school, middle school, and lower and upper secondary school. No children in the youngest bracket would have started school during 2020-2021 but those aged 6 in 2020 would have been in pre-school during wave 1 and then in first grade during wave 2 and 3. School in Sweden is compulsory from 6 to 15 years of age and about 85% of all 16–18-year-olds attend upper secondary school. About 90% of all Swedish 2-year-olds attend pre-school, with even higher rates for 4-5 year-olds(12).

Confounding variables

Weight and height were measured by standard anthropometric measurement techniques, and continuous BMI values (kg/m^2) were calculated, as BMI has been showed to be one of the major risk factors for severe COVID-19. Earlier studies on the same cohort have shown an association between BMI and cardiorespiratory fitness (CRF) in early adulthood and later risk of severe disease (11,13–15). There is also a known correlation between CRF, height and BMI at conscription and the probability of having children (16).

Morbidity at baseline

All medical diagnoses prior to conscription are recorded in the conscript registry. Illness that could have affected the ability or decision to have children, as well as later risk of severe COVID-19 was controlled for using ICD-codes (International Classification of Diseases) for respiratory disease, cardiovascular disease, diabetes, kidney disease and malignant cancers (16,17).

Socioeconomic indicators

Parental education was considered a proxy for socioeconomic position of the household. Using data from the LISA registry, it was based on the highest in the household and categorized as: low (up to 9 years), medium (upper secondary school diploma with ≤2 years at university) and high education (≥3 years at university). Data on home municipality was collapsed into three categories – large, medium and small towns and the municipalities nearby(18). Disposable family income was categorized into low, medium, and high income based on tertiles.

For peer review only

From LISA we also had information on profession. High risk occupation was defined a posteriori as those where the risk of hospitalisation due to COVID-19 was 0.28% and higher. This included health care personnel, bus drivers, restaurant workers, service personnel, industrial workers, social workers and primary school teachers (N=314 834).

Analytic sample

Originally comprising 1 949 891 conscripts, the cohort was reduced to 1 559 187 men after exclusions (Figure 1). 1 557 061 had information in LISA on children at home. Those who died during 2020 and until February 2021 (n=5 012) were censored in the analysis prior to each wave, giving an at risk population of 1 555 835 at the beginning of wave 1, 1 552 040 at wave 2 and 1 549 514 at wave 3. Characteristics of the population, together with crude outcome data, are shown in Table 1.

Figure 1 here

Table 1: Characteristics of the study population.

Conscription year	*1968-1975*	*1976-1985*	*1986-1995*	*1996-2005*	*All decades*
N in 2020	282 828	440 991	495 775	337 467	1 557 061
Age at conscription, mean (SD)	18.5 (0.65)	18.3 (0.82)	18.3 (0.76)	18.2 (0.58)	18.3 (0.73)
Age in 2020 mean (SD)	65.9 (1.94)	57.5 (2.98)	48.0 (3.00)	38.3 (2.91)	51.8 (9.89)
	Children at home (age in 2018)				
Child of any age	43 220 (15.3)	177 630 (40.3)	333 582 (67.3)	212 025 (62.8)	790 604 (50.8)
0-3 years (%)	292 (0.1)	3 151 (0.7)	38 873 (7.9)	111 926 (33.2)	154 242 (9.9)
4-6 years (%)	457 (0.2)	5 657 (1.3)	62 168 (12.5)	92 332 (27.4)	160 612 (10.3)
7-10 years (%)	1 275 (0.5)	16 576 (3.8)	124 800 (25.2)	79 418 (23.5)	222 069 (14.3)
11-15 years (%)	3 967 (1.4)	47 911 (10.9)	168 507 (34)	35 318 (10.5)	255 703 (16.4)
16-17 years (%)	3 597 (1.3)	35 946 (8.2)	68 234 (13.8)	5 549 (1.6)	113 326 (7.3)
18-20 years (%)	4 719 (1.7)	40 733 (9.2)	50 320 (10.2)	2 641 (0.8)	98 413 (6.3)
20 and older (%)	34 259 (12.1)	94 456 (21.4)	51 782 (10.4)	15 983 (4.7)	196 480 (12.6)
	Hospitalisations due to COVID-19				
N, March 2020-Sep 2021 (%)	2 261 (0.80)	3 179 (0.72)	2 455 (0.50)	760 (0.23)	8 655 (0.56)
With children at home (%)	426 (18.8)	1 318 (41.5)	1 616 (65.8)	463 (61)	3 823 (44.2)
	PCR confirmed infection with SARS-CoV-2				
N, March 2020-Sep 2021(%)	18 471 (6.5)	50 272 (11.4)	72 878 (14.7)	47 825 (14.2)	189 446 (12.2)
With children at home (%)	3 793 (20.5)	24 279 (48.3)	54 920 (75.4)	32 671 (68.3)	115 663 (61.1)

For peer review only

Outcome variables

Hospitalisation due to Covid-19

Using the Swedish personal identification number, the full sample was linked to the National Patient Register and the Swedish Intensive Care Registry. From these, all cases between March 2020 and September 2021 with a main diagnosis of ICD U071 for test verified infection with SARS-CoV-2 and U072 for clinically diagnosed COVID-19 were identified. Records with U071 or U072 as a secondary diagnosis were counted as cases if the main diagnosis was clinically related to COVID-19 (table S1). All hospital-care requiring illness was considered severe COVID-19. Register data based on Swedish hospital records have high validity (19).

Infection with COVID-19

Free PCR-testing began in summer 2020. Previously, testing was mainly done in hospitals. Therefore, data from this period is limited and not representative of the actual infection rates, as is seen in the comparison between testing and hospitalisations in Figure 2. All positive tests were to be registered in the Sminet registry according to the Swedish Communicable Diseases Act. Data from Sminet was extracted on September 21st, 2021 and covers all positives until that day.

Figure 2 here

Statistical analysis

The main independent variable was analysed as a binary variable in each age interval. Logistic regression was used to calculate the odds for hospitalisation and infection due to COVID-19 by this exposure category, adjusted for children in other age brackets, place of residence, income and profession in 2018, linear, quadratic, and cubic terms of BMI, as well as CRF, height, parental education and chronic disease at conscription. All regression models were adjusted for exact age at conscription and year of conscription examination, and thus indirectly for age in 2020. Because overall events of hospitalisation were rare, we used penalized likelihood estimation (Firth method) to reduce (potential) small-sample bias in maximum likelihood estimation

Statistical analyses were performed with SAS version 9.4 (SAS Institute, Cary, NC). Statistical significance was set at 0.05 (2-sided tests).

Ethics approval statement

The study conforms to the principles outlined in the Declaration of Helsinki. The Ethics Committee of the University of Gothenburg and Confidentiality Clearance at Statistics Sweden approved the study (EPN Reference numbers EPN 462-14 and 567-15; T174-15, T653-17, T196-17, T2019-05875, T 2020-01325, T2020-02420, T2021-00797, T2021-03310). The requirement for informed consent was waived by

For peer review only

the Ethics Committee of the University of Gothenburg for secondary analysis of existing data. The data was pseudonymized before being accessed by the study authors.

Results

Hospitalisations

Having a child of preschool age at home had a protective association with hospitalisation due to COVID-19 during the pandemic from March 2020 to July 2021 (Figure 3). This association is statistically significant in the first and third wave with OR=0.64 (95% CI 0.46-0.88) and 0.73 (0.58-0.91). During the second wave, no association could be seen (OR=1.06 (0.80-1.41)). In contrast, no associations between living with children of primary school age and hospitalisation were observed. Sharing the household with children 13 years of age or older conveyed an overall excess risk of hospitalisation.

Figure 3 here

None of the results was attenuated after adjustment for covariates (table S2). Exposures describing children of different age groups were not mutually exclusive, as many fathers live with more than one child. For the combined waves, the protective association between the youngest children and hospitalisation due to COVID-19 was strengthened to OR 0.54 (95% CI 0.42-0.71) when we considered a separate category of those with only pre-school aged children (table S3).

Infection

Living in a household with children over 5 was associated with an increased risk for infection with SARS-CoV-2. The same pattern can be seen for all waves, with odds ratios significantly higher for all age groups apart from the pre-school children who had a significant protective association (Figure 4) with OR=0.91 (95% CI 0.89-0.93). Wave specific results can be found in Supplementary table S4.

Figure 4 here

Adjustment for high-risk profession in the main analysis did not attenuate the effect estimates. To evaluate the effects of isolating with children not yet in compulsory school we examined the subset of men in high-risk occupations only: having younger children still gave a protective association with OR= 0.94 (95% CI 0.91-0.98). Again, when looking at those living with pre-school children only, the association was stronger with OR=0.73 (95% CI 0.69-0.77).

Discussion

For peer review only

In this paper we demonstrate a robust association between residing with children of pre-school age and a lower risk of severe COVID-19 during two of the three waves of the pandemic in Sweden.

The pattern for the older children varied, but no significant associations were seen at any time among those aged 6-12 years. Exposure to the age group 13-17 years was associated with a higher risk of severe COVID-19 during the second and third waves. Sharing a household with a teenager or young adult was associated with a higher risk of disease during all waves.

There were significantly higher odds for testing positive for SARS-CoV-2 associated with living with children 6 years and older, except for the age group 6-8 during the second wave. The age group 13-17 presents the largest risk for infection and includes both children who attended school (age 13-15) and who had distance learning (age 16-17). It has been shown that adolescents transmit COVID-19 disease similarly/comparably to adults in households, and the combination of slightly older children in open schools could explain this pattern (7,20–26). Unfortunately, the testing data does not include negative results, therefore it is not possible to analyse whether the infection rates partly mirror an increased testing frequency in certain groups.

The comparable studies from Scotland and England showed that having children at home (any age in England, 0-11 in Scotland) was not associated with increased risk of infection or severe disease in spring 2020. During the second wave risk of infection and COVID-19 related hospital admission was increased in the OpenSAFELY cohort but not in the Scottish cohort(7,8). This finding was attributed in part to the school closures during spring and the reopening after summer. Our results fit the same pattern but cannot be explained the same way, since the schools were open. An earlier Swedish study presents a similar small increase in infections among parents of lower-secondary school age children during the first wave(27).

High-risk profession did not affect the associations. With our smaller age brackets, we were able to single out associations in parents of children in pre-school, which were distinctly different from those with children of older ages and highlights a methodological strength of our study.

No national figures of school or pre-school attendance are available, but in the numbers of the official statistics bureau of Gothenburg (pop. 583 056) school attendance was distinctly lower in March 2020, and sick leave was higher during the pandemic compared to 2019. The possibility to self-isolate with younger children, together with more social distancing and better use of other protective measures in these families could of course contribute to the protective association, particularly considering that the effect was more pronounced in those with no older children. The Swedish pre-school teacher's union reported a large drop in attendance in March 2020. After March though, the majority returned to pre-school (28), making it unlikely that a large proportion of parents were isolating with their children. The fact that few of the pre-school fathers also lived with children older than 13 does limit their exposure to infection due to transmission in school, which also could contribute to the protective association. When the model is

For peer review only

unadjusted for older children the association is stronger with OR 0.85 (95% CI 0.83-0.87), but it is still significant in the main model where the presence or lack of older children is accounted for.

Our large study population, based on validated registry data and including key covariates reflecting the conscript's comorbidities and physical condition, is an important strength. However, since this is an observational study, we cannot rule out that unknown factors such as behavioural differences between those living with or without children of different ages influenced the results. The major limitation is that only men were included in this study. Furthermore, all information from the LISA registry is from 2018. However, only minor changes in these covariates can be expected during 2019-2020, except for the oldest children who might have left home during this period.

We were not able to include the effects of vaccination, but as the main part of our cohort were vaccinated from May 2021, the potential effect is restricted to the tapering off of wave 3, with very few cases overall.

Clinical and public health implications

The decision to keep mandatory schools open in Sweden constitute a comparison point. A recent review of studies trying to evaluate the effect of school closures on community transmission concluded that "The true independent effect of school closures from the first wave around the world may simply be unknowable"(29). The model calculations included in the review all had problems differentiating between NPI's implemented simultaneously. Closing schools was meant to control community transmission by limiting transmission between children and subsequently between children and parents. As our study shows, the odds ratio for infection was higher for men living with children of all age groups except those aged 2-5. This could be expected due to the greater number of contacts in school but is still comparable to transmission patterns where schools were closed during spring 2020.

The finding that living with children aged 2-5 was associated with lower risk of hospitalization due to COVID-19 does raise questions. If the effect is not entirely due to behavioural differences or parental health it could be speculated that simultaneous infection with other respiratory viruses more commonly occurring in this group compared to older children(30), such as rhinovirus, could be protective, as has been shown in vitro(31,32). Both wave 1 and wave 3 coincide with the months when the Swedish Social Insurance Agency normally distributes the most compensation for care of sick child (mainly due to cold viruses in early spring).

Conclusion

Young children seem to have played a minor part in the community transmission of COVID-19, even though pre-schools remained open in Sweden. As this study shows, adult men living in the same household as children of this age group had a lower risk both of infection and severe sickness. Having children between 6 and 12 years in the household was associated with a small increase in odds of

For peer review only

infection, but not with severe disease. Having teenagers in the household was associated with increased rates of infection as well as severe disease in their fathers. These associations are similar in magnitude to those reported in other settings where schools were closed.

For peer review only

Statements

Contributorship statement: AG, LL and MÅ initiated the project. AG and KM performed all statistical analyses. AG had main responsibility for writing the article. LL, MÅ, KM and MH contributed to the structure and content of the manuscript and have read and approved of the final draft. LL and MÅ share last authorship.

Competing interest statement: All authors have completed the Unified Competing Interest form (available on request from the corresponding author) and declare: no support from any other than above mentioned organization for the submitted work; no financial relationships with any organizations that might have an interest in the submitted work in the previous three years, no other relationships or activities that could appear to have influenced the submitted work.

Funding: This work was supported by the EpiLife-Teens Research Program (FORMAS-2012-00038), by the Swedish ALF-agreement (ALFGBG-720201 and ALFGBG-813511), the Swedish Research Council (02508, VRREG 2019-00193, 2020-05792) and Swedish Research Council for Health, Working Life and Welfare (2021-00304). The funding sources had no role in study design, collection, analysis and interpretation of data, the writing of the report, or the decision to submit the article for publication.

Data sharing statement: The data used in this study is available upon request from The Swedish Defence Conscription and Assessment Agency and the National Board of Health and Welfare.

Transparency statement: AG affirms that the manuscript is an honest, accurate, and transparent account of the study being reported and that no important aspects of the study have been omitted.

Dissemination to participants and related patient and public communities: We will make the findings of this article public with a press release and via media contacts.

For peer review only

References

1. Viner RM, Mytton OT, Bonell C, Melendez-Torres G, Ward J, Hudson L, et al. Susceptibility to SARS-CoV-2 infection among children and adolescents compared with adults: a systematic review and meta-analysis. JAMA pediatrics. 2021;175(2):143–56.

2. Ludvigsson JF. Systematic review of COVID-19 in children shows milder cases and a better prognosis than adults. Acta paediatrica. 2020;109(6):1088–95.

3. Cauchemez S, Ferguson NM, Wachtel C, Tegnell A, Saour G, Duncan B, et al. Closure of schools during an influenza pandemic. The Lancet infectious diseases. 2009;9(8):473–81.

4. Mangtani P. Impact of school closures on an influenza pandemic: scientific evidence base review. 2014;

5. Jackson C, Mangtani P, Hawker J, Olowokure B, Vynnycky E. The effects of school closures on influenza outbreaks and pandemics: systematic review of simulation studies. PloS one. 2014;9(5):e97297.

6. Utbildningsdepartementet. Förordning (2020:115) om utbildning på skolområdet och annan pedagogisk verksamhet vid spridning av viss smitta [Internet]. Mar 13, 2020. Available from: https://www.riksdagen.se/sv/dokument-lagar/dokument/svensk-forfattningssamling/forordning-2020115-om-utbildning-i-vissa_sfs-2020-115

7. Wood R, Thomson E, Galbraith R, Gribben C, Caldwell D, Bishop J, et al. Sharing a household with children and risk of COVID-19: a study of over 300 000 adults living in healthcare worker households in Scotland. Archives of disease in childhood. 2021;

8. Forbes H, Morton CE, Bacon S, McDonald HI, Minassian C, Brown JP, et al. Association between living with children and outcomes from covid-19: OpenSAFELY cohort study of 12 million adults in England. bmj. 2021;372.

9. Public Health Agency of Sweden. Bekräftade fall i Sverige – daglig uppdatering [Internet]. Public Health Agency of Sweden; Available from: https://www.folkhalsomyndigheten.se/smittskydd-beredskap/utbrott/aktuella-utbrott/covid-19/statistik-och-analyser/bekraftade-fall-i-sverige/

10. Af Geijerstam A, Mehlig K, Börjesson M, Robertson J, Nyberg J, Adiels M, et al. Fitness, strength and severity of COVID-19: a prospective register study of 1 559 187 Swedish conscripts. BMJ open. 2021;11(7):e051316.

11. Robertson J, Adiels M, Lissner L, Mehlig K, Af Geijerstam A, Lindgren M, et al. BMI in early adulthood is associated with severe COVID-19 later in life–a prospective cohort study of 1.5 million Swedish men. Obesity. 2022;

12. Förskola – Barn och grupper – Riksnivå [Internet]. Available from: https://www.skolverket.se/skolutveckling/statistik

13. Gao M, Piernas C, Astbury NM, Hippisley-Cox J, O'Rahilly S, Aveyard P, et al. Associations between body-mass index and COVID-19 severity in 6· 9 million people in England: a prospective, community-based, cohort study. The Lancet Diabetes & Endocrinology. 2021;

For peer review only

14. Hamer M, Gale CR, Kivimäki M, Batty GD. Overweight, obesity, and risk of hospitalization for COVID-19: A community-based cohort study of adults in the United Kingdom. Proceedings of the National Academy of Sciences. 2020;117(35):21011–3.

15. Clark A, Jit M, Warren-Gash C, Guthrie B, Wang HH, Mercer SW, et al. Global, regional, and national estimates of the population at increased risk of severe COVID-19 due to underlying health conditions in 2020: a modelling study. The Lancet Global Health. 2020;8(8):e1003–17.

16. Barclay K, Kolk M. The influence of health in early adulthood on male fertility. Population and Development Review. 2020;46(4):757–85.

17. Glazer CH, Bonde JP, Eisenberg ML, Giwercman A, Hærvig KK, Rimborg S, et al. Male infertility and risk of nonmalignant chronic diseases: a systematic review of the epidemiological evidence. In Thieme Medical Publishers; 2017. p. 282–90.

18. Sveriges kommuner och regioner. Kommungruppsindelning [Internet]. Available from: https://skr.se/skr/tjanster/kommunerochregioner/faktakommunerochregioner/kommungruppsindelning.2051.html

19. Ludvigsson JF, Andersson E, Ekbom A, Feychting M, Kim JL, Reuterwall C, et al. External review and validation of the Swedish national inpatient register. BMC public health. 2011;11(1):1–16.

20. Madewell ZJ, Yang Y, Longini IM, Halloran ME, Dean NE. Household Transmission of SARS-CoV-2: A Systematic Review and Meta-analysis. JAMA network open. 2020;3(12):e2031756–e2031756.

21. Soriano-Arandes A, Gatell A, Serrano P, Biosca M, Campillo F, Capdevila R, et al. Household SARS-CoV-2 transmission and children: a network prospective study. Clinical Infectious Diseases: an Official Publication of the Infectious Diseases Society of America. 2021;

22. Li F, Li YY, Liu MJ, Fang LQ, Dean NE, Wong GW, et al. Household transmission of SARS-CoV-2 and risk factors for susceptibility and infectivity in Wuhan: a retrospective observational study. The Lancet Infectious Diseases. 2021;21(5):617–28.

23. Brandal LT, Ofitserova TS, Meijerink H, Rykkvin R, Lund HM, Hungnes O, et al. Minimal transmission of SARS-CoV-2 from paediatric COVID-19 cases in primary schools, Norway, August to November 2020. Eurosurveillance. 2021;26(1):2002011.

24. Goldstein E, Lipsitch M, Cevik M. On the Effect of Age on the Transmission of SARS-CoV-2 in Households, Schools, and the Community. The Journal of infectious diseases. 2021;223(3):362–9.

25. Telle K, Jørgensen SB, Hart R, Greve-Isdahl M, Kacelnik O. Secondary attack rates of COVID-19 in Norwegian families: A nation-wide register-based study. European journal of epidemiology. 2021;1–8.

26. Dattner I, Goldberg Y, Katriel G, Yaari R, Gal N, Miron Y, et al. The role of children in the spread of COVID-19: Using household data from Bnei Brak, Israel, to estimate the relative susceptibility and infectivity of children. PLoS computational biology. 2021;17(2):e1008559.

27. Vlachos J, Hertegård E, Svaleryd HB. The effects of school closures on SARS-CoV-2 among parents and teachers. Proceedings of the National Academy of Sciences. 2021;118(9).

For peer review only

28. Fock C. Coronatapp: 9 av 10 förskolebarn hölls hemma. Förskolan [Internet]. 2020 Jul 6; Available from: https://www.lararen.se/forskolan/coronaviruset/coronatapp-9-av-10-forskolebarn-holls-hemma

29. Walsh S, Chowdhury A, Braithwaite V, Russell S, Birch JM, Ward JL, et al. Do school closures and school reopenings affect community transmission of COVID-19? A systematic review of observational studies. BMJ Open. 2021 Aug 1;11(8):e053371.

30. Monto AS. Epidemiology of viral respiratory infections. The American journal of medicine. 2002;112(6):4–12.

31. Dee K, Goldfarb DM, Haney J, Amat JA, Herder V, Stewart M, et al. Human rhinovirus infection blocks SARS-CoV-2 replication within the respiratory epithelium: implications for COVID-19 epidemiology. Journal of Infectious Diseases. 2021;

32. Kuitunen I, Artama M, Haapanen M, Renko M. Rhinovirus spread in children during the COVID-19 pandemic despite social restrictions—A nationwide register study in Finland. Journal of Medical Virology. 2021;93(10):6063–7.

For peer review only

Legends

Figure 1: Creation of analytical sample.

Table 1: Characteristics of the study population.

Figure 2: Weekly admissions to hospital due to COVID-19 in Sweden and weekly registered PCR-verified infections between March 2020 and September 2020. Note that tests were not widely available until July 2020.

Figure 3: Associations between children in the household and hospitalisation due to COVID-19 (n=1 557 061). Odds ratios with 95% CI. Model controlled for children in other age groups, age, baseline BMI, CRF, height, chronic morbidity, parental education, income, profession and place of residence in 2018.

Figure 4: Associations between children in the household and testing positive for SARS-CoV-2 March 2020-September 2021 (n=1 557 061). Odds ratios with 95% CI. Model controlled for conscript's age, BMI, CRF, height, chronic morbidity, parental education and place of residence in 2018.

For peer review only

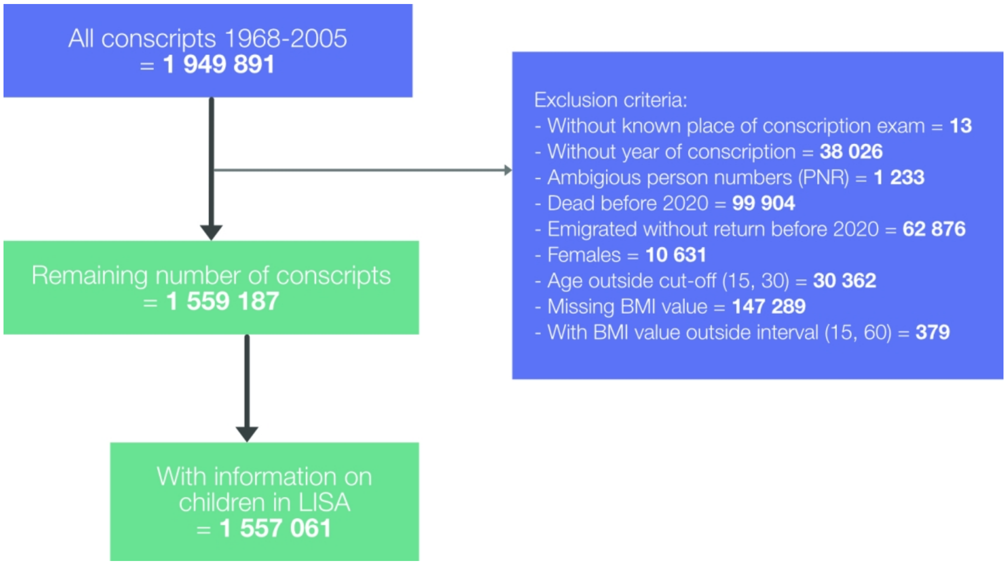

136x76mm (300 x 300 DPI)

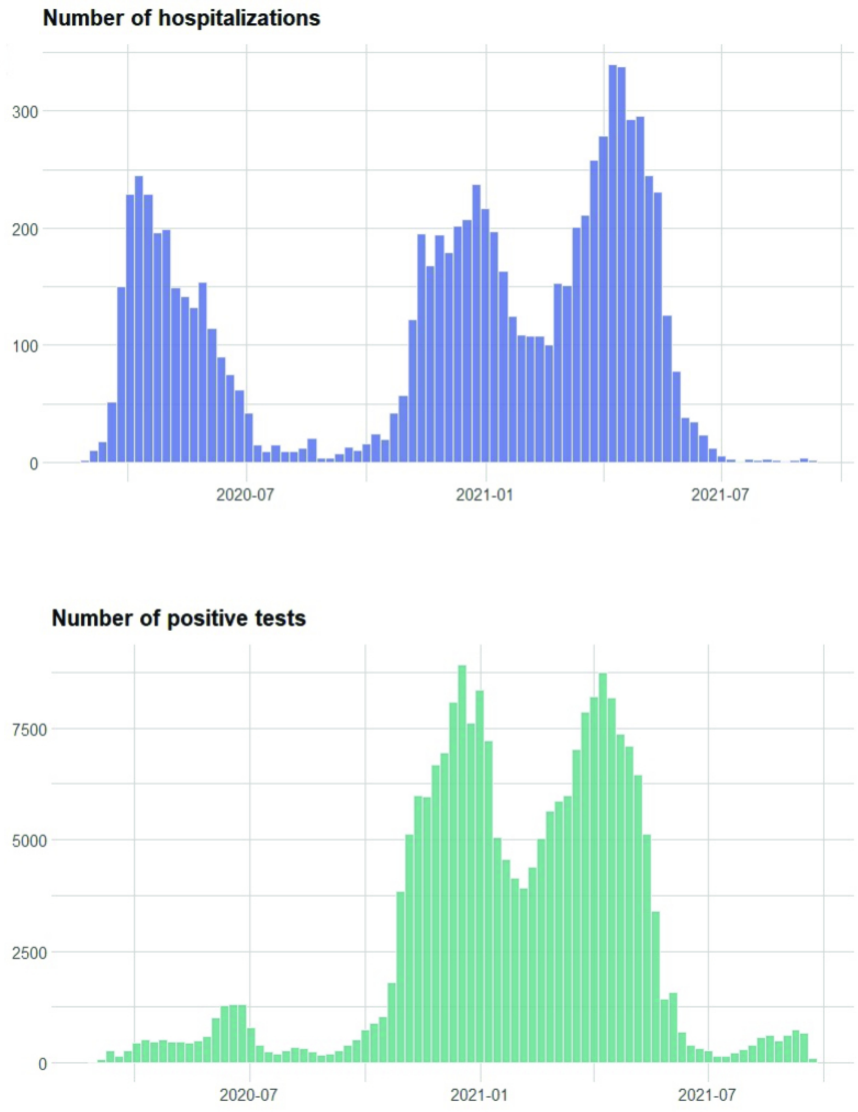

72x92mm (300 x 300 DPI)

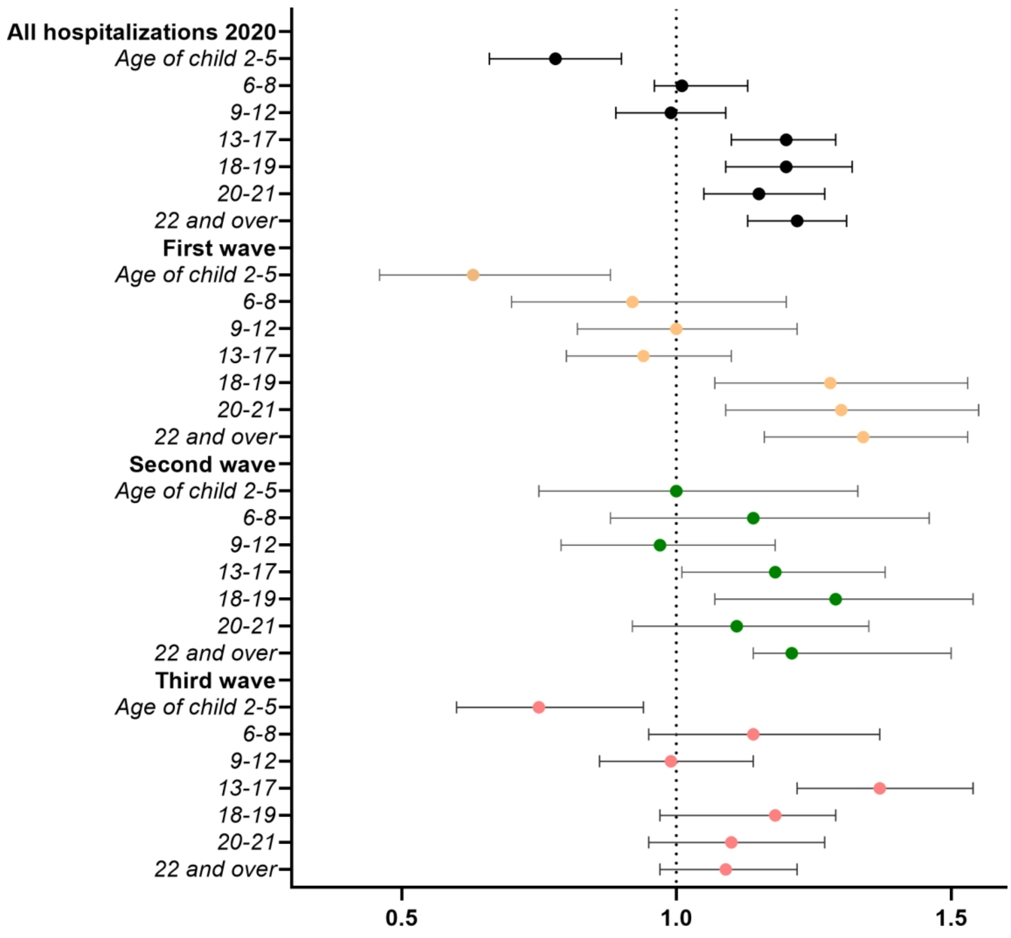

166x152mm (300 x 300 DPI)

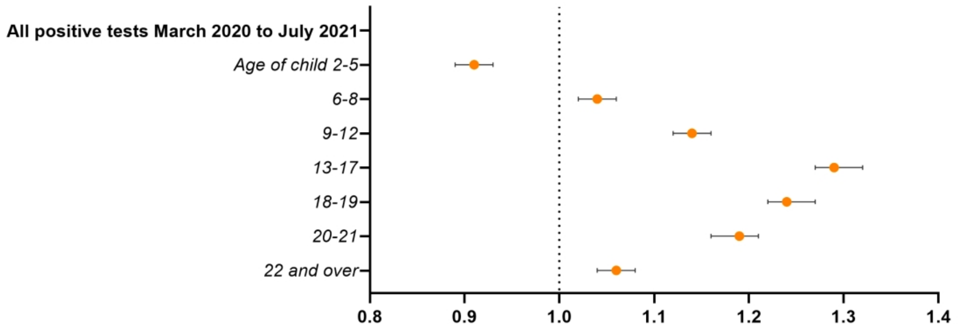

215x79mm (300 x 300 DPI)

Table S1. Included main diagnoses divided by categories when COVID-19 is secondary diagnosis.

Included categories	ICD-codes
**COVID-related symptoms**	
Cough	R05
Abnormalities of breathing	R06
Pain in throat and chest	R07
Other symptoms and signs involving the circulatory and respiratory system	R09
Dizziness and giddiness	R42
Fever of other or unknown origin	R50
Headache	R51
Malaise and fatigue	R53
Syncope and collapse	R55
**Upper and lower respiratory tract infections**	
Acute nasopharyngitis	J00
Acute tonsillitis	J03
Acute upper respiratory infections of multiple and unspecified sites	J06
Influenza due to other identified influenza virus	J10
Other viral pneumonia	J128
Viral pneumonia, unspecified	J129
Pneumonia due to Streptococcus pneumoniae	J13
Bacterial pneumonia, not elsewhere classified	J15
Pneumonia due to other specified infectious organisms	J168
Pneumonia in diseases classified elsewhere	J17
Pneumonia, unspecified organism	J18
Unspecified acute lower respiratory infection	J22
Coronavirus infection, unspecified	B342
Other viral infections of unspecified site	B348
Viral infection, unspecified	B349
Coronavirus as the cause of diseases classified elsewhere	B972
Other and unspecified infectious diseases	B99
**Respiratory disorders**	
Pulmonary embolism	I26
Acute respiratory distress syndrome	J80
Pulmonary edema	J81
Pleural effusion not elsewhere classified	J90
Respiratory failure, not elsewhere classified	J96
Respiratory disorders in diseases classified elsewhere	J99
**Obstructive Airway Diseases**	
Acute bronchitis due to other specified organisms	J208
Acute bronchitis, unspecified	J209
Acute bronchiolitis due to other specified organisms	J218
Acute bronchiolitis, unspecified	J219
Other chronic obstructive pulmonary disease	J44
Asthma	J45
**Cardiac diseases**	
Viral carditis	B332
Chronic ischemic heart disease	I25
Acute pericarditis	I30
Pericarditis in diseases classified elsewhere	I32
Acute myocarditis, unspecified	I40
Myocarditis in diseases classified elsewhere	I41
Atrial fibrillation and flutter	I48
Heart failure	I50
Abnormalities of heart beat	R00

For peer review only

**Kidney disorders**	
Acute kidney failure	N17
Chronic kidney disease	N18
Unspecified kidney failure	N19
**Electrolyte disorders**	
Other disorders of fluid, electrolyte and acid-base balance	E87

For peer review only

Table S2: Result of hospitalization analysis. OR, 95% CI. Basic model includes adjusting for age in 2020 and children of other ages. Reference for each age bracket is children in other brackets or no children at all.

Age of child in 2020	*Basic model*	*Adjusted for baseline CRF*	*Adjusted for baseline BMI, height, morbidity*	*Adjusted for parental education, income, region*	*Adjusted for profession*	*Full model*
	All hospitalizations March 2020 – September 2021					
N	1 557 061	1 142 222	1 557 061	1 420 206	1 557 061	1 027 123
n of cases	8 655	6 731	8 655	7 497	8 655	5 765
2-5	**0.74** (0.66-0.83)	**0.72** (0.62-0.84)	**0.76** (0.68-0.85)	**0.78** (0.70-0.88)	**0.75** (0.66-0.84)	**0.78** (0.66-0.90)
6-8	0.95 (0.86-1.05)	1.02 (0.89-1.16)	0.96 (0.87-1.06)	0.99 (0.90-1.10)	0.95 (0.86-1.05)	1.01 (0.95-1.13)
9-12	0.98 (0.90-1.06)	0.94 (0.84-1.02)	0.99 (0.92-1.08)	1.03 (0.95-1.11)	0.98 (0.91-1.06)	0.99 (0.89-1.10)
13-17	**1.16** (1.09-1.24)	**1.16** (1.07-1.25)	**1.18** (1.10-1.26)	**1.21** (1.13-1.29)	**1.16** (1.08-1.23)	**1.19** (1.10-1.29)
18-19	**1.16** (1.07-1.25)	**1.16** (1.06-1.27)	**1.17** (1.08-1.27)	**1.18** (1.09-1.29)	**1.16** (1.07-1.26)	**1.20** (1.09-1.32)
20-22	1.09 (0.99-1.18)	1.10 (0.99-1.20)	**1.10** (1.01-1.19)	**1.14** (1.05-1.25)	1.08 (0.99-1.17)	**1.15** (1.04-1.27)
22 and over	**1.15** (1.08-1.21)	**1.17** (1.09-1.24)	**1.15** (1.08-1.21)	**1.20** (1.12-1.28)	**1.14** (1.08-1.21)	**1.20** (1.13-1.30)
	Wave 1 (March-September 2020)					
n of cases	2 430	1 884	2 430	2 096	2 430	1 612
2-5	**0.71** (0.57-0.89)	**0.60** (0.44-0.82)	**0.73** (0.58-0.91)	**0.75** (0.60-0.94)	**0.72** (0.57-0.89)	**0.64** (0.46-0.88)
6-8	0.86 (0.70-1.05)	0.86 (0.66-1.12)	0.87 (0.72-1.06)	0.91 (0.74-1.12)	0.87 (0.71-1.06)	0.91 (0.70-1.19)
9-11	0.91 (0.78-1.07)	0.91 (0.75-1.10)	0.93 (0.79-1.08)	0.99 (0.84-1.16)	0.91 (0.78-1.07)	1.00 (0.82-1.22)
12-17	0.94 (0.83-1.07)	0.90 (0.78-1.05)	0.96 (0.84-1.09)	1.00 (0.87-1.14)	0.94 (0.83-1.07)	0.94 (0.80-1.10)
18-19	1.16 (0.99-1.35)	**1.23** (1.04-1.46)	1.17 (1.00-1.37)	**1.18** (1.00-1.41)	1.15 (0.99-1.35)	**1.26** (1.05-1.51)
20-22	1.16 (0.99-1.35)	**1.22** (1.03-1.45)	1.17 (1.00-1.37)	**1.24** (1.05-1.46)	1.15 (0.99-1.34)	**1.30** (1.09-1.55)
22 and over	**1.24** (1.11-1.38)	**1.24** (1.10-1.40)	**1.24** (1.12-1.38)	**1.33** (1.18-1.51)	**1.24** (1.11-1.38)	**1.32** (1.15-1.51)
	Wave 2 (September 2020-February 2021)					
n of cases	2 575	2 041	2 575	2 182	2 575	1 707
2-5	**0.80** (0.64-0.99)	0.94 (0.72-1.24)	0.82 (0.66-1.03)	0.86 (0.68-1.08)	0.80 (0.64-1.00)	1.06 (0.80-1.41)
6-8	0.93 (0.76-1.12)	1.04 (0.81-1.33)	0.94 (0.77-1.15)	0.99 (0.80-1.21)	0.93 (0.76-1.13)	1.15 (0.90-1.48)
9-11	0.99 (0.85-1.16)	0.90 (0.75-1.11)	1.00 (0.86-1.17)	1.04 (0.88-1.22)	0.99 (0.85-1.16)	0.96 (0.78-1.17)
12-17	1.11 (0.97-1.26)	1.15 (0.99-1.32)	1.14 (1.00-1.29)	**1.17** (1.02-1.33)	1.12 (0.99-1.26)	**1.22** (1.04-1.41)
18-19	**1.20** (1.03-1.40)	1.16 (0.98-1.38)	**1.21** (1.04-1.41)	**1.28** (1.09-1.50)	**1.20** (1.03-1.40)	**1.28** (1.07-1.52)
20-22	1.01 (0.86-1.18)	1.02 (0.85-1.21)	1.02 (0.87-1.20)	1.08 (0.91-1.28)	1.01 (0.86-1.18)	1.11 (0.92-1.35)

For peer review only

22 and over	**1.17** (1.05-1.31)	**1.19** (1.07-1.35)	**1.17** (1.05-1.30)	**1.27** (1.12-1.42)	**1.16** (1.05-1.29)	**1.31** (1.15-1.50)
			Wave 3 (February 2021-July 2021)			
n of cases	3 650	2 806	3 650	3 219	3 650	2 446
2-5	**0.73** (0.62-0.87)	**0.69** (0.55-0.86)	**0.75** (0.64-0.89)	**0.76** (0.64-0.90)	**0.73** (0.62-0.87)	**0.73** (0.58-0.91)
6-8	1.01 (0.88-1.17)	1.10 (0.92-1.31)	1.03 (0.90-1.19)	1.05 (0.90-1.21)	1.01 (0.88-1.17)	1.14 (0.95-1.37)
9-11	1.02 (0.91-1.14)	0.97 (0.84-1.11)	1.04 (0.93-1.16)	1.04 (0.92-1.17)	1.02 (0.91-1.14)	0.99 (0.86-1.14)
12-17	**1.33** (1.21-1.46)	**1.34** (1.20-1.50)	**1.35** (1.23-1.48)	**1.36** (1.23-1.50)	**1.33** (1.21-1.46)	**1.35** (1.21-1.52)
18-19	**1.14** (1.01-1.28)	1.11 (0.97-1.27)	**1.15** (1.02-1.30)	1.13 (1.00-1.29)	**1.14** (1.01-1.29)	1.12 (0.97-1.30)
20-22	1.08 (0.96-1.23)	1.06 (0.92-1.20)	1.10 (0.97-1.24)	1.13 (0.99-1.29)	1.08 (0.96-1.23)	1.08 (0.93-1.26)
22 and over	1.07 (0.97-1.17)	1.09 (0.98-1.21)	1.07 (0.97-1.17)	1.07 (0.96-1.18)	1.07 (0.99-1.16)	1.07 (0.95-1.20)

Table S3: Associations between children in the household and hospitalization due to COVID-19. Model controlled for age, baseline BMI, CRF, height, chronic morbidity, parental education, income, profession and place of residence in 2018. OR, 95% CI.

	Only children 2-5	***Only children 6-12***	***Only children 13-22***	***Only Children 6-22***
N	66 385	119 397	382 277	698 938
n of cases	120	426	2 504	3 690
March 2020-July 2021	**0.54** (0.42-0.71)	0.95 (0.84-1.08)	**1.28** (1.21-1.36)	**1.30** (1.22-1.39)
Wave 1	**0.49** (0.29-0.82)	**0.74** (0.57-0.98)	**1.30** (1.16-1.46)	**1.22** (1.08-1.38)
Wave 2	0.60 (0.36-1.00)	0.78 (0.59-1.02)	**1.34** (1.20-1.50)	**1.37** (1.21-1.54)
Wave 3	**0.57** (0.40-0.82)	1.18 (0.99-1.40)	**1.24** (1.13-1.36)	**1.31** (1.19-1.44)

For peer review only

Table S4: Result of infection analysis. OR, 95% CI. Basic model includes adjusting for age in 2020 and children of other ages. Reference for each age bracket is children in other brackets or no children at all.

Age of child in 2020	*Basic model*	*Adjusted for baseline CRF*	*Adjusted for baseline BMI, height, morbidity*	*Adjusted for parental education, income*	*Adjusted for profession*	*Full model*
			**All test positives**			
N	1 557 061	1 142 222	1 557 061	1 420 206	1 557 061	1 027 123
n of cases	189 446	135 503	189 446	179 211	189 446	126 946
2-5	**1.04** (1.02-1.06)	**0.98** (0.96-0.99)	**1.04** (1.02-1.05)	**0.95** (0.94-0.97)	**1.04** (1.02-1.06)	**0.91** (0.89-0.93)
6-8	**1.13** (1.11-1.15)	**1.01** (1.07-1.12)	**1.12** (1.11-1.14)	**1.06** (1.04-1.07)	**1.13** (1.11-1.15)	**1.04** (1.01-1.06)
9-11	**1.26** (1.24-1.27)	**1.22** (1.20-1.24)	**1.25** (1.23-1.27)	**1.17** (1.15-1.18)	**1.25** (1.24-1.27)	**1.14** (1.12-1.16)
12-17	**1.43** (1.41-1.45)	**1.41** (1.39-1.43)	**1.42** (1.41-1.44)	**1.31** (1.29-1.33)	**1.43** (1.41-1.45)	**1.29** (1.27-1.32)
18-19	**1.34** (1.32-1.36)	**1.33** (1.30-1.35)	**1.33** (1.31-1.36)	**1.25** (1.23-1.27)	**1.34** (1.32-1.36)	**1.24** (1.22-1.27)
20-22	**1.30** (1.28-1.33)	**1.29** (1.26-1.31)	**1.29** (1.27-1.32)	**1.20** 81.18-1.22)	**1.30** (1.28-1.33)	**1.19** (1.16-1.21)
22 and over	**1.16** (1.15-1.18)	**1.20** (1.18-1.22)	**1.16** (1.14-1.18)	**1.02** (1.00-1.03)	**1.16** (1.14-1.18)	**1.06** (1.04-1.08)
			**Wave 1**			
n of cases	10 937	8 034	10 937	10 110	10 937	7 355
2-5	0.95 (0.88-1.02)	0.92 (0.84-1.02)	0.95 (0.88-1.02)	**0.90** (0.83-0.97)	0.95 (0.88-1.02)	**0.89** (0.80-0.98)
6-8	0.96 (0.90-1.03)	0.97 (0.89-1.06)	0.96 (0.89-1.03)	**0.92** (0.85-0.98)	0.96 (0.90-1.03)	0.94 (0.85-1.02)
9-11	1.01 (0.95-1.07)	1.01 (0.94-1.08)	1.01 (0.95-1.07)	0.96 (0.90-1.02)	1.01 (0.95-1.07)	0.96 (0.89-1.04)
12-17	**1.20** (1.14-1.26)	**1.17** (1.10-1.24)	**1.19** (1.13-1.26)	**1.13** (1.07-1.19)	**1.19** (1.13-1.25)	**1.10** (1.04-1.17)
18-19	**1.34** (1.26-1.43)	**1.38** (1.28-1.48)	**1.34** (1.26-1.43)	**1.26** (1.18-1.35)	**1.34** (1.25-1.43)	**1.30** (1.21-1.40)
20-22	**1.30** (1.21-1.39)	**1.31** (1.22-1.42)	**1.29** (1.20-1.38)	**1.23** (1.15-1.32)	**1.29** (1.20-1.38)	**1.23** (1.14-1.34)
22 and over	**1.17** (1.10-1.23)	**1.22** (1.15-1.30)	**1.16** (1.10-1.23)	**1.08** (1.02-1.15)	**1.16** (1.10-1.23)	**1.13** (1.05-1.21)
			**Wave 2**			
n of cases	81 923	59 038	81 923	77 276	81 923	55 142
2-5	1.01 (0.99-1.04)	**0.96** (0.93-0.98)	1.01 (0.98-1.03)	**0.92** (0.90-0.95)	1.01 (0.99-1.04)	**0.88** (0.85-0.91)
6-8	**1.06** (1.03-1.08)	1.02 (0.99-1.05)	**1.05** (1.03-1.08)	0.99 (0.97-1.01)	**1.06** (1.03-1.08)	**0.96** (0.93-0.99)
9-11	**1.20** (1.17-1.22)	**1.15** (1.12-1.18)	**1.20** (1.17-1.22)	**1.11** (1.09-1.14)	**1.20** (1.17-1.22)	**1.08** (1.05-1.11)
12-17	**1.37** (1.35-1.40)	**1.35** (1.32-1.37)	**1.36** (1.34-1.39)	**1.25** (1.23-1.28)	**1.37** (1.34-1.39)	**1.24** (1.21-1.26)
18-19	**1.28** (1.25-1.31)	**1.26** (1.22-1.30)	**1.27** (1.24-1.30)	**1.20** (1.17-1.23)	**1.27** (1.25-1.31)	**1.18** (1.15-1.22)

20-22	**1.27** (1.24-1.31)	**1.26** (1.22-1.30)	**1.26** (1.23-1.29)	**1.17** (1.14-1.20)	**1.27** (1.24-1.30)	**1.16** (1.13-1.20)
22 and over	**1.17** (1.15-1.20)	**1.21** (1.18-1.24)	**1.17** (1.14-1.19)	**1.03** (1.01-1.05)	**1.17** (1.15-1.20)	**1.06** (1.03-1.09)
			**Wave 3**			
n of cases	96 586	68 431	96 586	91 825	96 586	64 449
2-5	**1.06** (1.04-1.08)	1.00 (0.97-1.03)	**1.06** (1.03-1.08)	0.99 (0.97-1.01)	**1.06** (1.04-1.08)	**0.94** (0.91-0.97)
6-8	**1.18** (1.16-1.21)	**1.16** (1.12-1.18)	**1.18** (1.15-1.20)	**1.12** (1.09-1.14)	**1.18** (1.16-1.21)	**1.10** (1.07-1.13)
9-11	**1.28** (1.26-1.31)	**1.25** (1.23-1.28)	**1.28** (1.26-1.31)	**1.20** (1.18-1.22)	**1.28** (1.26-1.30)	**1.18** (1.16-1.21)
12-17	**1.42** (1.40-1.45)	**1.41** (1.38-1.44)	**1.42** (1.40-1.44)	**1.32** (1.30-1.34)	**1.42** (1.40-1.45)	**1.31** (1.28-1.34)
18-19	**1.33** (1.30-1.36)	**1.31** (1.28-1.35)	**1.32** (1.29-1.35)	**1.24** (1.22-1.27)	**1.33** (1.30-1.36)	**1.24** (1.21-1.27)
20-22	**1.28** (1.25-1.31)	**1.25** (1.22-1.29)	**1.27** (1.24-1.30)	**1.18** (1.15-1.21)	**1.28** (1.25-1.31)	**1.17** (1.13-1.20)
22 and over	**1.13** (1.10-1.15)	**1.17** (1.14-1.19)	**1.12** (1.10-1.15)	0.99 (0.98-1.02)	**1.13** (1.10-1.15)	**1.04** (1.01-1.06)

For peer review only
